# Genetic variants of *CYP2C19* and *CYP2C18* and their clinical implications in the Saudi population^☆^

**DOI:** 10.1016/j.jtumed.2026.03.003

**Published:** 2026-03-20

**Authors:** Abdullah Alkattan, Ahmed Alhajri, Yousef Almutairi, Mahmoud Kandeel, Abdulla Al-Taher

**Affiliations:** a Department of Research, Training and Development, Assisting Deputyship for Primary Health Care, Ministry of Health, Riyadh, KSA; b Department of Biomedical Sciences, College of Veterinary Medicine, King Faisal University Al-Ahsa, KSA; c Department of Pharmacy, King Abdulaziz National Guard Health Affairs, Ministry of National Guard, Al-Ahsa, KSA; d General Administration of Medical Supply, Ministry of Health, Riyadh, KSA; e Department of Pharmacology, Faculty of Veterinary Medicine, Kafrelsheikh University, Kafrelsheikh, Egypt

**Keywords:** *CYP2C18* gene, *CYP2C19* gene, Drug effectiveness, Drug toxicity, Genetic variants, Pharmacogenomics, جين CYP2C19, جين CYP2C18, علم الصيدلة الجينية, المتغيرات الجينية, فعالية الدواء, سمية الدواء

## Abstract

**Background:**

At least 500 genetic polymorphisms associated with the CYP2C19 and CYP2C18 enzymes have been identified, yet few polymorphisms have been measured in the Saudi population. Therefore, this study was aimed at determining the frequencies of numerous unmeasured *CYP2C19* and *CYP2C18* genetic variations, and their clinical and pharmacological implications, in KSA.

**Research design and methods:**

A multicenter cross-sectional study was conducted to determine the frequency of 141 CYP2C19 and CYP2C18 genetic variants. Blood samples from 374 Saudi adults were genotyped. GenomeStudio software was used for genotype calling for all samples. Bioinformatics analyses were performed for variant annotation. We excluded the rs4244285 variant since our main objective was to determine the frequency of numerous unmeasured or poorly documented CYP2C19 and CYP2C18 genetic variants in KSA. Including rs12769205 mitigated the haplotype analysis concern. Both rs4244285 and rs12769205 are CYP2C19∗2 key variations linked to loss of function.

**Results:**

Of the 141 variants associated with the CYP2C19 and CYP2C18 genes measured in 374 participants, 53 were detected, and the other 88 were not detected. Five detected variants had high frequencies: rs7067866, rs7916649, rs4917623, rs12248560, and rs11188072. Several detected variants were associated with clinical effects and appeared to influence drug metabolism.

**Conclusions:**

This study investigated the presence of many previously undescribed CYP2C19 and CYP2C18 genetic variants in the Middle Eastern population. Tens of previously unmeasured CYP2C19 and CYP2C18 variants were identified in the Saudi population. Our findings have potential to contribute to pharmacogenomics and personalized medicine, and to support clinical decision-making.

## Highlights

• Limited CYP2C19 and CYP2C18 polymorphisms have been measured in the Saudi population.
• This multicenter study examined 141 genetic variants in 374 Saudis.
• Of the 141 variants investigated, 53 were detected, including five highly prevalent functional variants.
• Highly frequent variants have been associated with altered pharmacokinetics and pharmacodynamics of common drugs.
• Four of the observed *CYP2C18* variants in Saudis were associated with the ultra-rapid CYP2C19 metabolizer phenotype.

## الملخص

**أهداف البحث:**

تم التعرف على ما لا يقل عن 500 شكل جيني متعدد مرتبط بإنزيمي CYP2C19 وCYP2C18، إلا أن عددا قليلا من هذه الأشكال الجينية تم قياسه في المجتمع السعودي. لذلك، هدفت هذه الدراسة إلى تحديد انتشار العديد من التغيرات الجينية غير المقاسة لإنزيمي CYP2C19 وCYP2C18، وآثارها السريرية والدوائية، في المملكة العربية السعودية.

**طرق البحث:**

أُجريت دراسة مقطعية متعددة المراكز لتحديد معدل انتشار 141 متغيرًا جينيًا لإنزيمي CYP2C19 وCYP2C18. تم تحليل النمط الجيني لعينات دم من 374 بالغا سعوديا. استُخدم برنامج GenomeStudio لتحديد النمط الجيني لجميع العينات. كما أُجريت تحليلات المعلوماتية الحيوية لشرح المتغيرات. تم استبعاد المتغير rs4244285 من التحليل، لأن هدفنا الرئيسي كان تحديد معدل انتشار العديد من المتغيرات الجينية غير المقاسة أو الموثقة بشكل غير كافٍ لجيني *CYP2C19 وCYP2C18* في المملكة العربية السعودية. ورغم احتمال تأثر تحليل النمط الفرداني، فقد تم التخفيف من هذا التخوف جزئيا بإضافة المتغير rs12769205. يُعد كل من rs4244285 وrs12769205 من المتغيرات الرئيسية المعروفة لجين *CYP2C19*∗2 المرتبطة بفقدان وظيفة هذا الجين.

**النتائج:**

من بين 141 متغيرا مرتبطا بجيني *CYP2C19 وCYP2C18* تم قياسها لدى 374 مشاركا، تم الكشف عن 53 متغيرا، بينما لم يتم الكشف عن الـ 88 المتبقية. خمس متغيرات جينية تم رصدها كانت ذات ترددات عالية: rs7067866، rs7916649، rs4917623، rs12248560، وrs11188072. ارتبطت عدة متغيرات جينية تم رصدها بتأثيرات سريرية، ويبدو أنها تؤثر على استقلاب الدواء.

**الاستنتاجات:**

بحثت هذه الدراسة وجود العديد من المتغيرات الجينية غير الموصوفة سابقا لإنزيمي CYP2C19 وCYP2C18 لدى سكان الشرق الأوسط. تم تحديد عشرات المتغيرات الجينية غير المقاسة سابقا لإنزيمي CYP2C19 وCYP2C18 لدى السكان السعوديين. تُسهم نتائجنا في علم الصيدلة الجينية والطب الشخصي، وتدعم اتخاذ القرارات السريرية.

## Introduction

Pharmacogenomics, an important branch of pharmacology, involves the study of genetic variations among diverse populations, including possible effects on drug kinetics and dynamics. Genetic variations among diverse populations are associated with hyper-response, normal-response, hypo-response, non-response, or hypersensitivity to specific drugs. Therefore, pharmacogenomic aspects warrant substantial consideration in medicine.^1^^,^^2^ Numerous mutations in genes that encode metabolic enzymes, such as CYP2C19, influence the pharmacokinetics of multiple medications. CYP2C19, a cytochrome P450 enzyme, oxidizes several widely used medications, such as clopidogrel, tamoxifen, voriconazole, phenytoin, diazepam, escitalopram, progesterone, and omeprazole.^1^, ^2^, ^3^, ^4^, ^5^ The CYP2C19 enzyme is composed of 490 amino acids and is encoded by the *CYP2C19* gene. Mutations in the *CYP2C19* gene affect the metabolism of multiple drugs. In addition, because the *CYP2C18* and *CYP2C19* genes share >80 % sequence identity and are located at the same locus, several genetic mutations of *CYP2C1*8 might also influence the metabolism of CYP2C19 enzyme substrates.^6^, ^7^, ^8^

Although multiple drugs are recognized CYP2C19 and CYP2C18 substrates, their degree of metabolism varies depending on the nature of their chemical structures. The CYP2C19 enzyme, and potentially its cognate CYP2C18, are involved in metabolizing several drugs, including clopidogrel, warfarin, lansoprazole, escitalopram, and voriconazole. 7, 9 The *CYP2C19* and *CYP2C18* genes have more than 500 known variants; however, many of these variants are rare.^10^ The *CYP2C19∗1* and *CYP2C18∗1* alleles are recognized as wild type and encode normal active forms of CYP2C19 and CYP2C18. Common variants include *CYP2C19*∗2, *CYP2C19*∗3, *CYP2C19*∗17, rs2860840 (*CYP2C18*), rs2901783 (*CYP2C18)*, and rs7917985 (*CYP2C18*).^11^ The *CYP2C19∗17* mutant allele encodes a more active version of the CYP2C19 enzyme, whereas the *CYP2C19∗2* and *CYP2C19∗3* mutant alleles result in the production of an inactive CYP2C19 enzyme.^11^ A significantly high prevalence of *CYP2C19∗2* and *CYP2C19∗3* has been reported in Chinese, Filipino, Thai, Malaysian, Japanese, and Indigenous Australian groups.^12^ By contrast, Europeans and Africans have high rates of the *CYP2C19∗*17 allele.^12^

Several studies have indicated that Saudi inhabitants of particular regions frequently carry the *CYP2C19∗2*, *CYP2C19*∗17, and *CYP2C19*∗1B alleles, whereas very few people carry the *CYP2C19∗3*, *CYP2C19∗6*, *CYP2C19∗8*, *CYP2C19∗9*, *CYP2C19∗13*, and *CYP2C19∗15* alleles.^6^^,^^11^^,^^13^ In addition, the *CYP2C19∗4*, *CYP2C19∗7*, *CYP2C19∗10*, *CYP2C19∗11*, *CYP2C19∗12*, and *CYP2C19∗14* alleles have not been detected among Saudis.^6^^,^^13^ Previous Saudi investigations were conducted in the cities of Riyadh and Abha, yet limited knowledge is available regarding *CYP2C19* variants in other Saudi areas with populations belonging to various Arabic tribes. Additionally, to our knowledge, *CYP2C18* genetic variants have not previously been studied in KSA. Although Saudi studies have measured the common CYP2C19 variants, several other variants associated with *CYP2C19* and its cognate *CYP2C18* gene have not been measured.

Hence, this study was intended to fill the data gap regarding other unmeasured *CYP2C19* and *CYP2C18* genetic variants among inhabitants of KSA, and to investigate the potential clinical and pharmacological implications. Because *CYP2C19* and *CYP2C18* share a locus and exhibit high sequence homology, they were investigated together.

The Alahsa Metropolitan Area was chosen as the study location, because of its diverse population; urban growth due primarily to the presence of lands owned by the Saudi Arabian Oil Company (ARAMCO) and the National Guard; and a substantial population increase from 500,000 inhabitants in 1990 to 1.24 million in 2017.^14^

## Participants and methods

### Research design and participants

This multicenter cross-sectional study conducted in the Alahsa Metropolitan Area, KSA, from December 2022 to September 2023, involved healthy Saudi adults randomly selected from 22 primary healthcare centers and five governmental hospitals across the four principal cities of the Alahsa region: Al-Hofuf, Al-Mobarraz, Al-Oyoun, and Al-Omran. In Roasoft Inc. software, the minimum sample size was calculated to be 303 participants, on the basis of the estimated adult population in the Alahsa region in 2020 (N = 800,000), a confidence level of 95 %, and an assumed proportion of 0.27 derived from prior studies in KSA suggesting that approximately 27 % of the population has *CYP2C19* gene polymorphisms.^10^^,^^12^ Notably, although the calculated sample size was adequate for detecting common variant associations, it was underpowered for detecting rare variant associations (minor allele frequency [MAF] <1 %).

### DNA extraction and quantification

Blood samples (4 mL) were collected from each participant and stored at −20 °C before genotype analysis. To extract DNA from the collected blood samples, we used a commercial kit (QIAamp DNA mini kit, Qiagen, USA). After extraction, the DNA was quantified with PicoGreen assays and a Synergy HTX instrument (Promega, cat. E2670). DNA quality and purity were evaluated with a NanoDrop spectrophotometer. Absorbance measurements at 260 nm were used to quantify the DNA. DNA samples with a ratio of absorbance at 260 to 280 nm between 1.7 and 2.0 were considered to be relatively pure and of good quality. These measurements indicated the concentration and purity of the DNA samples.

### DNA hybridization and scanning

An Infinium global screening array v 3.0 was used for genotyping of markers. According to the manufacturer's instructions, DNA was hybridized to a BeadChip, then scanned with an iScan® system. The bead locations on the BeadChip and quantification of the signal intensity were determined with downloaded BeadChip decode map (DMAP) files. The generated intensity data (∗.idat) files were combined with bead pool manifest (∗.bpm) files containing information on the single-nucleotide polymorphism (SNP)/probe content on the BeadChip and cluster files – also called ensemble graphics toolkit (∗.egt) files – which contain reference information for each interrogated locus. Finally, the these files were combined and converted to genotype call (∗.gtc) files, which were imported into GenomeStudio software for analysis.

### Data analysis

For data quality control, the GenTrain score, call frequency, number of calls, number of no calls, and ChiTest100 were measured. In addition, GenomeStudio version 2.0 software was used to modify the clusters, normalize data, and call genotypes for all samples. In GenomeStudio, a project was created with ∗.idat files containing the xy location and intensity data. The ∗.bpm file containing the bead ID and sequence information, and the ∗.egt file containing the genotyping cluster positions were used to accelerate the project creation process. These files were imported into GenomeStudio, and the project creation wizard was followed.

GenomeStudio was used to evaluate the samples. The main sample metric was the call rate, which is the percentage of all variants and insertion-deletion mutations (indels) successfully genotyped for a given sample. Any sample with a call rate <96 % was excluded. The call rate provided the starting point for quality control of assay or sample performance. The sample metric of logR deviation (logR dev), or the deviation in the normalized intensity value, was used to indicate sample contamination. The main SNP metric was the call frequency; the percentage of samples for which a genotype was called for a specific SNP. Another SNP metric, the GenTrain score, reflected how well GenomeStudio identified groups of alleles in a training data set. An ideal GenTrain score of 1 would have 3 clusters with equal R at 0, 0.5, and 1 norm Theta.

Moreover, we used ChiTest100 to calculate the *p*-value from a chi-squared test of Hardy–Weinberg equilibrium (HWE). The HWE test assesses whether the observed genotype frequencies for a given SNP in the study population are consistent with those expected under HWE. A high *p*-value indicates a high probability that the observed deviations from expected HWE frequencies are due to random chance, therefore, the data are consistent with the population being in HWE, and the SNP is probably of good quality and accurately genotyped. After samples and SNPs were evaluated, and data quality control was completed, a final report containing the allele calls was exported in variant call format (∗.vcf).

For bioinformatics analysis, we used BCFtools in bioinformatics pipelines for various applications, namely, CADD, DANN, regSNP-intron, VEST, and SIFT, to annotate the detected genetic variants.^15^^,^^16^ Furthermore, Haploview version 4.1 was used to analyze the linkage disequilibrium (LD) and HWE between all detected *CYP2C19* SNPs among study participants. The constructed haplotypes were visualized through an LD plot generated with Haploview.^17^^,^^18^ To measure LD among the genetic variants, we determined the D′, r^2^, and logarithm of the odds (LOD).

## Results

A total of 374 blood specimens were collected across various locations in the Alahsa area. The mean participant age was 40.1 ± 13.8 years, and approximately 53 % of participants were female (*n* = 199). The DNA samples were successfully isolated and met the quality assurance standards. Table S1 shows 146 genetic markers of *CYP2C19* and *CYP2C18* genes, and their chromosomal positions, GenTrain scores, call frequencies, numbers of calls, and numbers of no calls. We were able to measure 141 *CYP2C19* and *CYP2C18* variants. Most variants had high GenTrain scores (>0.8) and insignificant ChiTest100 *p*-values. In addition, the sample call rate was very high: more than 98 % of samples had a score of 1.

Table 1 lists the 53 identified *CYP2C19* and *CYP2C18* variants among the 141 tested variants, along with their star alleles and genotypes (homozygous dominant, homozygous recessive, or heterozygous). Some of these variants with high MAF (>20 %), including rs7916649, rs4917623, rs2901783, rs7915414, rs11528090, rs4494250, rs2860840, rs12248560, and rs4388808, have been associated with alterations in the pharmacokinetics and pharmacodynamics of commonly prescribed drugs, such as omeprazole, clopidogrel, nevirapine, tamoxifen, voriconazole, warfarin, and valproic acid. Additionally, five variants (rs4917623, rs4494250, rs12248560, rs4388808, and rs11592737) have been associated with various health disorders, acting either as risk factors or protective factors. These disorders include breast cancer, chronic obstructive pulmonary disease, endometriosis, endometrial thickness, tetralogy of Fallot, major depressive disorder, hypertension, type 2 diabetes mellitus, Alzheimer's disease, and female infertility (Table 2 and Table S2). Moreover, Table 3 shows the predictive functional consequences of the detected variants, according to computational tools. According to their DANN and SIFT scores, six (n = 6) variants, rs1126545, rs17879685, rs17885179, rs59636573, rs60181876, and rs770829708, were associated with major deterioration in CYP2C19 and CYP2C18 enzymatic activity and possibly with health issues. However, the other measured variants did not show significant in silico enzymatic deterioration.

**Table 1:** MAF and genotype frequencies of *CYP2C19* and *CYP2C18* gene variants detected among Saudi participants (n = 53).

Participant	SNP	Star allele, nitrogenous base change	MAF	Homozygous genotype for minor allele, N (%)	Heterozygous genotype for minor allele, N (%)	Homozygous genotype for wild-type allele, N (%)
1.	rs12769205	*CYP2C19*∗2, A > G	14.17	13 (3.48 %)	80 (21.39 %)	281 (75.13 %)
2.	rs12571421	*CYP2C19*∗2, A > G	11.36	8 (2.14 %)	69 (18.45 %)	297 (79.41 %)
3.	rs145119820	*CYP2C19*∗2, G > A	0.80	0 (0 %)	6 (1.6 %)	368 (98.4 %)
4.	rs7916649	*CYP2C19*∗2B, G > A	46.66	77 (20.59 %)	195 (52.14 %)	102 (27.27 %)
5.	rs770829708	*CYP2C19*∗2F, G > A	0.13	0 (0 %)	1 (0.27 %)	372 (99.73 %)
6.	rs17878459	*CYP2C19*∗3, G > A	0.67	0 (0 %)	5 (1.34 %)	369 (98.66 %)
7.	rs17879685	*CYP2C19*∗13, C > T	0.13	0 (0 %)	1 (0.27 %)	373 (99.73 %)
8.	rs3814637	*CYP2C19*∗15, C > T	7.91	2 (0.54 %)	55 (14.75 %)	316 (84.72 %)
9.	rs12248560	*CYP2C19*∗17, C > T	20.99	11 (2.94)	135 (36.1 %)	228 (61 %)
10.	rs11188072	*CYP2C19*∗17, C > T	20.99	11 (2.94 %)	135 (36.1 %)	228 (60.96 %)
11.	rs11188059	*CYP2C18* G > A	4.55	0 (0 %)	34 (9.09 %)	340 (90.91 %)
12.	rs7902257	*CYP2C19*∗27, G > A	2.28	1 (0.27 %)	15 (4.01 %)	358 (95.72 %)
13.	rs111490789	*CYP2C19*∗28, C > A	0.41	0 (0 %)	3 (0.81 %)	367 (99.19 %)
14.	rs4417205	*CYP2C19*∗35, C > G	14.17	13 (3.48 %)	80 (21.39 %)	281 (75.13 %)
15.	rs17885179	*CYP2C19*∗39, A > C	0.40	0 (0 %)	3 (0.8 %)	371 (99.2 %)
16.	rs28399513	*CYP2C19*, T > A	12.17	10 (2.67 %)	71 (18.98 %)	293 (78.34 %)
17.	rs11528090	*CYP2C19*, T > G	21.43	17 (4.58 %)	125 (33.69 %)	228 (61.73 %)
18.	rs12768009	*CYP2C19*, G > A	12.17	10 (2.67 %)	71 (18.98 %)	293 (78.34 %)
19.	rs2860840	*CYP2C18*, C > T	21.12	16 (4.27 %)	126 (33.68 %)	232 (62.03 %)
20.	rs1126545	*CYP2C18*, C > T	11.36	8 (2.1 %)	69 1 (8.45 %)	297 (79.4 %)
21.	rs4986894	*CYP2C19*∗2C, T > C	11.36	8 (2.14 %)	69 (18.45 %)	297 (79.41 %)
22.	rs12268020	*CYP2C19*, C > T	20.99	11 (2.94 %)	135 (36.1 %)	228 (60.96 %)
23.	rs4917623	*CYP2C19*, T > C	42.78	68 (18.18 %)	184 (49.2 %)	122 (32.62 %)
24.	rs11592737	*CYP2C19*, A > G	20.99	11 (2.94 %)	135 (36.1 %)	228 (60.96 %)
25.	rs7067866	*CYP2C19*, G > T	46.79	77 (22.59 %)	196 (52.41 %)	101 (27.01 %)
26.	rs7917985	*CYP2C18*, C > A	23.53	16 (4.28 %)	144 (38.5 %)	214 (57.22 %)
27.	rs2901783	*CYP2C18*, A > G	22.06	22 (5.88 %)	121 (32.35 %)	231 (61.76 %)
28.	rs4494250	*CYP2C19*, G > A	21.26	17 (4.55 %)	125 (33.42 %)	232 (62.03 %)
29.	rs4388808	*CYP2C19*, A > G	20.99	16 (4.28 %)	125 (33.42 %)	233 (62.3 %)
30.	rs11188092	*CYP2C19*, A > C	20.72	10 (2.67 %)	135 (36.1 %)	229 (61.23 %)
31.	rs7085563	*CYP2C18*, T > A	16.58	15 (4.01 %)	94 (25.13 %)	265 (70.86 %)
32.	rs2260946	*CYP2C19*, T > C	14.71	13 (3.48 %)	84 (22.46 %)	277 (74.06 %)
33.	rs7900135	*CYP2C18*, A > G	14.04	13 (3.48 %)	79 (21.12 %)	282 (75.4 %)
34.	rs932809	*CYP2C18*, C > T	14.04	13 (3.48 %)	79 (21.12 %)	282 (75.4 %)
35.	rs3740367	*CYP2C18*, G > A	4.81	1 (0.27 %)	34 (9.09 %)	339 (90.64 %)
36.	rs117140172	*CYP2C18*, G > A	2.01	1 (0.27 %)	13 (3.48 %)	360 (96.26 %)
37.	rs1326830	*CYP2C18*, C > A	2.01	0 (0 %)	15 (4.01 %)	359 (95.99 %)
38.	rs17884832	*CYP2C19*, T > G	6.95	2 (0.53 %)	48 (12.83 %)	324 (86.63 %)
39.	rs17879992	*CYP2C19*, T > C	7.37	2 (0.54 %)	51 (13.67 %)	321 (85.79 %)
40.	rs187091323	*CYP2C19*, A > G	4.58	1 (0.27 %)	32 (8.63 %)	341 (91.11 %)
41.	rs4917612	*CYP2C19*, C > G	9.09	6 (1.6 %)	56 (14.97 %)	312 (83.42 %)
42.	rs733115	*CYP2C19*, G > T	6.95	2 (0.53 %)	48 (12.83 %)	324 (86.63 %)
43.	rs941890	*CYP2C19*, G > A	2.54	1 (0.27 %)	17 (4.55 %)	356 (95.19 %)
44.	rs150579865	*CYP2C18*, G > T	0.53	0 (0 %)	4 (1.07 %)	370 (98.93 %)
45.	rs60181876	*CYP2C18*, C > T	0.67	0 (0 %)	5 (1.34 %)	369 (98.66 %)
46.	rs59636573	*CYP2C18*, G > T	0.13	0 (0 %)	1 (0.27 %)	373 (99.73 %)
47.	rs113164681	*CYP2C19*, C > T	0.94	0 (0 %)	7 (1.87 %)	367 (98.13 %)
48.	rs17878739	*CYP2C19*, T > C	0.94	0 (0 %)	7 (1.87 %)	367 (98.13 %)
49.	rs17878649	*CYP2C19*, G > A	0.27	0 (0 %)	2 (0.53 %)	372 (99.47 %)
50.	rs141417293	*CYP2C19*, C > A	0.27	0 (0 %)	2 (0.53 %)	372 (99.47 %)
51.	rs143612134	*CYP2C19*, T > C	0.27	0 (0 %)	2 (0.53 %)	372 (99.47 %)
52.	rs150790215	*CYP2C19*, G > A	0.80	0 (0 %)	6 (1.6 %)	368 (98.4 %)
53.	rs7915414	*CYP2C19*, G > A	22.01	22 (5.98 %)	120 (32.07 %)	232 (61.96 %)

**Table 2:** Effects of the most frequently detected (MAF >20 %) *CYP2C19* and *CYP2C18* gene polymorphisms on human health and the metabolism of several drugs.

Participant	Variant	Gene	MAF	Predicted enzymatic activity	Drugs	Effects of SNPs on drugs	Clinical implications
1.	rs7916649	*CYP2C19*	46.66	Inactive	Valproic acid and clopidogrel	Effects on valproic acid: increased risk of valproic acid toxicity.	NA
						Effects on clopidogrel: Increased risk of recurrent cardiac events due to treatment failure	
2.	rs4917623	*CYP2C19*	42.78	Decreased	Tamoxifen and nevirapine	Effects on tamoxifen: Enhanced anti-cancer efficacy.	The *CYP2C19* rs4917623 heterozygous genotype is more strongly associated with breast cancer (odds ratio = 1.38) than the wild-type homozygous genotype.
						Effects on nevirapine: rs4917623 allele might be associated with elevated trough plasma concentrations of nevirapine, and might lead to enhanced efficacy as well as adverse effects.	
3.	rs2901783	*CYP2C18*	22.06	Unconfirmed	Clopidogrel and warfarin	Effects on clopidogrel: rs2901783 variant is significantly associated with a lower clopidogrel efficacy than the referenced allele.	NA
						Effects on warfarin: rs2901783 is associated with accelerated achievement of a therapeutic INR.	
4.	rs7915414	*CYP2C19*	22.01	Unconfirmed	Clopidogrel	Effects on clopidogrel: rs7915414 variant is significantly associated with lower clopidogrel efficacy than the referenced allele.	NA
5.	rs11528090	*CYP2C19*	21.43	Unconfirmed; Potentially associated with decreased enzymatic activity	Nevirapine	Effects on nevirapine: rs11528090 allele might be associated with elevated trough plasma concentrations of nevirapine, and might lead to enhanced efficacy as well as adverse effects.	NA
6.	rs4494250	*CYP2C19*	21.26	Unconfirmed; Potentially associated with decreased enzymatic activity	Anti-HIV drugs (NNRTIs and PIs)	Effects on anti-HIV drugs (NNRTIs and PIs): carriers of the rs4494250 allele might have enhanced anti-HIV treatment efficacy by reducing viral load, if treated with NNRTI and PI drugs.	The rs4494250 allele might be associated with greater breast cancer risk than the wild-type allele.
							The rs4494250 allele is associated with enhanced risk of elevated diastolic blood pressure.
							The rs4494250 minor allele might be associated with greater chronic obstructive pulmonary disease risk than the wild-type allele.
7.	rs2860840	*CYP2C18*	21.12	Unconfirmed; potentially associated with increased enzymatic activity	Omeprazole, voriconazole, and warfarin	Effects on omeprazole: rs2860840 allele is associated with treatment failure and might necessitate increased doses.	NA
						Effects on voriconazole: rs2860840 allele is associated with treatment failure and might necessitate increased doses.	
						Effects on warfarin: rs2860840 allele is associated with diminished warfarin efficacy due to rapid metabolism.	
8.	rs12248560	*CYP2C19*	20.99	Increased	Clopidogrel, prasugrel, and tamoxifen	Effects on clopidogrel and prasugrel: carriers of the rs12248560 T allele have a significantly higher rate of hyper-response to clopidogrel and higher rates of bleeding complications than carriers of the CC genotype. Effects on tamoxifen: Women with breast cancer carrying the rs12248560 variant might have diminished response to tamoxifen.	Women carrying the rs12248560 T allele (CT and TT genotypes) have diminished risk of endometriosis and breast cancer, potentially in association with enhanced estradiol catabolism. Pregnant individuals carrying the rs12248560 T allele (CT and TT genotypes) might have elevated risk of delivering newborns with tetralogy of Fallot. Carriers of the rs12248560 T allele have elevated risk of hypertension and type-2 diabetes.
9.	rs11592737	*CYP2C19*	20.99	Unconfirmed; potentially associated with normal enzymatic activity	NA	NA	Women carrying the rs11592737 GG genotype have greater risk of endometriosis and infertility than those carrying the rs11592737 A allele.
							Women with the rs11592737 GG genotype have greater risk of infertility and endometriosis than those carrying the rs11592737 A allele.
10.	rs4388808	*CYP2C19*	20.99	Unconfirmed; potentially associated with decreased enzymatic activity	Nevirapine	Effects on nevirapine: rs4388808 G allele might be associated with elevated trough plasma concentrations of nevirapine and might lead to enhanced efficacy as well as adverse effects.	Carriers of the rs4388808 G allele have a significantly lower amyloid-β load in the frontal, inferior temporal, and posterior cingulate cortices in the brain than AA genotype carriers. Therefore, the rs4388808 G allele is recognized as a protective genetic factor against Alzheimer's disease.

NA: No relevant data or information is available. NNRTIs and PIs: Non-nucleoside reverse transcriptase inhibitors and protease inhibitors.

**Table 3:** Functional consequences of the detected variants (n = 53).

Participant	Variant ID	Gene	Sequence ontology	Protein sequence change	SIFT score^a^	CADD score^b^	DANN score^c^	regSNP-intron Ω	VEST score ¶	Predicted enzymatic activity
1.	rs111490789	*CYP2C19*	Non-coding	–	–	0.681	0.2773	–	–	Unconfirmed
2.	rs11188059	*CYP2C18*	Non-coding	–	–	7.594	0.5633	0.5	–	Increased
3.	rs11188072	*CYP2C19*	Non-coding	–	–	0.25	0.3745	–	–	Increased
4.	rs11188092	*CYP2C19*	Non-coding	–	–	1.331	0.3358	0.67	–	Unconfirmed
5.	rs1126545	*CYP2C18*	Missense	T385M	**0.034**	**15.28**	**0.9723**	–	0.318	Inactive
6.	rs113164681	*CYP2C19*	Non-coding	–	–	0.479	0.383	–	–	Unconfirmed
7.	rs11528090	*CYP2C19*	Non-coding	–	–	1.56	0.3714	0.14	–	Unconfirmed; Potentially associated with decreased enzymatic activity
8.	rs11592737	*CYP2C19*	Non-coding	–	–	0.313	0.7489	0.63	–	Unconfirmed; potentially associated with normal enzymatic activity
9.	rs117140172	*CYP2C18*	Non-coding	–	–	2.138	0.4199	0.25	–	Unconfirmed
10.	rs12248560	*CYP2C19*	Regulatory	–	–	0.233	0.3703	–	–	Increased
11.	rs12268020	*CYP2C19*	Non-coding	–	–	0.269	0.3836	0.67	–	Unconfirmed; potentially associated with normal enzymatic activity
12.	rs12571421	*CYP2C19*	Non-coding	–	–	3.321	0.7417	0.55	–	Inactive
13.	rs12768009	*CYP2C19*	Non-coding	–	–	1.909	0.7394	0.46	–	Unconfirmed; Potentially associated with decreased enzymatic activity
14.	rs12769205	*CYP2C19*	Non-coding	–	–	1.03	0.6396	0.26	–	Inactive
15.	rs1326830	*CYP2C18*	Non-coding	–	–	0.193	0.2623	–	–	Unconfirmed
16.	rs141417293	*CYP2C19*	Non-coding	–	–	2.043	0.1969	0.45	–	Unconfirmed
17.	rs143612134	*CYP2C19*	Non-coding	–	–	3.585	0.6413	0.77	–	Unconfirmed
18.	rs145119820	*CYP2C19*	Missense	V113I	1	0.002	0.8678	–	0.176	Decreased
19.	rs150579865	*CYP2C18*	Non-coding	–	–	0.46	0.2657	0.66	–	Unconfirmed
20.	rs150790215	*CYP2C19*	Non-coding	–	–	3.571	0.3808	0.52	–	Unconfirmed
21.	rs17878459	*CYP2C19*	Missense	E92D	0.365	1.085	0.6485	–	–	Inactive
22.	rs17878649	*CYP2C19*	Non-coding	–	–	2.796	0.2572	0.73	–	Unconfirmed
23.	rs17878739	*CYP2C19*	Non-coding	–	–	0.173	0.2972	–	–	Unconfirmed
24.	rs17879685	*CYP2C19*	Missense	R410C	**0.037**	**16.42**	0.08931	–	0.264	Unconfirmed
25.	rs17879992	*CYP2C19*	Non-coding	–	–	1.02	0.6592	0.52	–	Unconfirmed
26.	rs17884832	*CYP2C19*	Non-coding	–	–	5.644	0.7492	0.39	–	Unconfirmed
27.	rs17885179	*CYP2C19*	Missense	E122A	0.281	**19.47**	**0.9636**	**-**	**0.52**	Unconfirmed; Potentially associated with decreased enzymatic activity
28.	rs187091323	*CYP2C19*	Non-coding	–	–	1.662	0.09841	0.48	–	Unconfirmed
29.	rs2260946	*CYP2C19*	Non-coding	–	–	1.639	0.3275	0.24	–	Unconfirmed
30.	rs28399513	*CYP2C19*	Non-coding	–	–	0.418	0.2191	0.25	–	Unconfirmed
31.	rs2860840	*CYP2C18*	Non-coding	–	–	1.686	0.5899	–	–	Unconfirmed; Potentially associated with increased enzymatic activity
32.	rs2901783	*CYP2C18*	Non-coding	–	–	0.182	0.4646	0.32	–	Unconfirmed
33.	rs3740367	*CYP2C18*	Non-coding	–	–	0.204	0.2274	–	–	Unconfirmed
34.	rs3814637	*CYP2C19*	Non-coding	–	–	4.773	0.5647	–	–	Inactive
35.	rs4388808	*CYP2C19*	Non-coding	–	–	4.562	0.8193	0.26	–	Unconfirmed; Potentially associated with decreased enzymatic activity
36.	rs4417205	*CYP2C19*	Non-coding	–	–	5.274	0.6617	0.21	–	Unconfirmed
37.	rs4494250	*CYP2C19*	Non-coding	–	–	1.011	0.3958	0.32	–	Unconfirmed; Potentially associated with decreased enzymatic activity
38.	rs4917612	*CYP2C19*	Non-coding	–	–	0.054	0.3323	0.09	–	Unconfirmed
39.	rs4917623	*CYP2C19*	Non-coding	–	–	0.243	0.3293	0.97	–	Decreased
40.	rs4986894	*CYP2C19*	Non-coding	–	–	2.632	0.5392	–	–	Unconfirmed
41.	rs59636573	*CYP2C18*	Missense	V330L	**0.001**	**18.8**	**0.9871**	–	**0.666**	Unconfirmed
42.	rs60181876	*CYP2C18*	Missense	T299I	**0.025**	**23.6**	**0.9979**	–	0.453	Unconfirmed
43.	rs7067866	*CYP2C19*	Non-coding	–	–	0.628	0.1727	–	–	Unconfirmed; Potentially associated with increased enzymatic activity
44.	rs7085563	*CYP2C18*	Non-coding	–	–	1.291	0.2497	0.38	–	Unconfirmed
45.	rs733115	*CYP2C19*	N/K	–	–	3.121	0.4313	–	–	Unconfirmed
46.	rs770829708	*CYP2C19*	Missense	D341N	**0**	**26**	**0.9992**	–	0.39	Decreased
47.	rs7900135	*CYP2C18*	Non-coding	–	–	8.62	0.8819	0.23	–	Unconfirmed
48.	rs7902257	*CYP2C19*	Regulatory	–	–	0.186	0.7151	–	–	Decreased
49.	rs7915414	*CYP2C19*	Non-coding	–	–	0.408	0.3888	0.79	–	Unconfirmed
50.	rs7916649	*CYP2C19*	Non-coding	–	–	1.204	0.2696	0.28	–	Inactive
51.	rs7917985	*CYP2C18*	Non-coding	–	–	0.432	0.6275	0.51	–	Unconfirmed; potentially associated with normal enzymatic activity
52.	rs932809	*CYP2C18*	Non-coding	–	–	2.372	0.3141	0.67	–	Unconfirmed
53.	rs941890	*CYP2C19*	N/K	–	–	0.014	0.1292	–	–	Unconfirmed

Ω regSNP-intron: This score is only for intronic variants. Scores <0.05 indicate a damaging protein, whereas scores between 0.05 and 0.1 indicate a possibly damaging protein.

¶ VEST (variant effect scoring tool) score: This score is only for exonic variants. Scores range from 0.0 to 1.0. A score >0.5 indicates a possible deleterious variant.

N/K: Unknown.

a SIFT (sorting intolerant from tolerant) score: This score is only for exonic variants. Scores range from 0.0 to 1.0. A score ≤0.05 indicates a possible deleterious variant.

b CADD (combined annotation-dependent depletion) score: Scores ≥10 indicate that the variant is among the 10 % most deleterious substitutions affecting the human genome.

c DANN (deleterious annotation of genetic variants with neural network) score: Scores range from 0 to 1. A score >0.5 indicates a possible deleterious variant.

Table 4 presents the LD among the 53 observed genetic variants according to their D′, r^2^, and LOD values. The variants rs7900135, rs932809, rs7085563 (*CYP2C18* variants), and rs12768009 (a *CYP2C19* variant) were strongly associated with, and inherited together with, the rs12769205 (*CYP2C19*∗2E) variant related to CYP2C19 enzyme inactivity. Furthermore, the rs7917985 (a *CYP2C18* variant), rs11188072, rs11188092, rs11592737, and rs12268020 variants correlated with the rs12248560 variant (also called *CYP2C19*∗17 and associated with CYP2C19 ultra-activity). In addition, the rs28399513 and rs12768009 variants were associated with the rs12571421 variant (also called *CYP2C19*∗2 and associated with inactive CYP2C19). Moreover, rs7067866, a highly detected *CYP2C19* variant (MAF = 46.8 %), was strongly associated with rs7916649 (*CYP2C19*∗2B).

**Table 4:** D′, logarithm of the odds, correlation coefficient, and 95 % confidence intervals for SNP pairs.

Participant	SNP-1	SNP-2	D′	LOD	r^2^	95 %CI_lower_	95 %CI_upper_
1.	rs7900135	rs12769205	1	105.53	0.989	0.97	1
2.	rs7917985	rs12248560	1	101.62	0.863	0.97	1
3.	rs932809	rs12769205	1	105.53	0.989	0.97	1
4.	rs7085563	rs12769205	0.988	83.1	0.812	0.94	1
5.	rs1126545	rs12768009	1	85.24	0.926	0.96	1
6.	rs11188072	rs12248560	1	126.27	1	0.98	1
7.	rs113164681	rs111490789	1	7.57	1	0.6	1
8.	rs111490789	rs17878739	1	7.57	1	0.6	1
9.	rs3814637	rs17884832	1	58.15	0.872	0.94	1
10.	rs4986894	rs12768009	1	85.24	0.926	0.96	1
11.	rs7067866	rs7916649	1	162.46	0.995	0.98	1
12.	rs17884832	rs17879992	1	62.48	0.941	0.95	1
13.	rs4388808	rs11528090	1	123.11	0.976	0.98	1
14.	rs12571421	rs28399513	1	85.24	0.926	0.96	1
15.	rs12768009	rs12571421	1	85.24	0.926	0.96	1
16.	rs2860840	rs4494250	1	127.22	0.992	0.98	1
17.	rs4417205	rs28399513	1	80.73	0.839	0.96	1
18.	rs3740367	rs187091323	1	41.93	0.964	0.92	1
19.	rs11188092	rs11592737	1	120.76	0.984	0.98	1
20.	rs11188092	rs12268020	1	120.76	0.984	0.98	1
21.	rs12248560	rs11188092	1	120.76	0.984	0.98	1
22.	rs932809	rs28399513	0.987	77.86	0.826	0.94	1
23.	rs12768009	rs12769205	1	80.73	0.839	0.96	1
24.	rs12768009	rs28399513	1	98.89	1	0.98	1
25.	rs11592737	rs12268020	1	126.27	1	0.98	1
26.	rs7917985	rs11592737	1	101.62	0.863	0.97	1
27.	rs11188072	rs11592737	1	126.27	1	0.98	1
28.	rs12248560	rs11592737	1	126.27	1	0.98	1
29.	rs59636573	rs17879685	1	3.01	1	0.28	1
30.	rs11188072	rs12268020	1	126.27	1	0.98	1
31.	rs12248560	rs12268020	1	126.27	1	0.98	1
32.	rs17884832	rs733115	1	67.54	1	0.96	1
33.	rs7902257	rs941890	1	27.95	0.892	0.87	1

**D′**: value of D′ between loci.

**LOD**: log of the likelihood odds ratio, a measure of confidence in the value of D′.

**r^2^**: correlation coefficient between loci.

**95 %CI_lower_**: 95 % confidence interval of the lower bound on D′.

**95 %CI_upper_**: 95 % confidence interval of the upper bound on D′.

**Figure 1:**
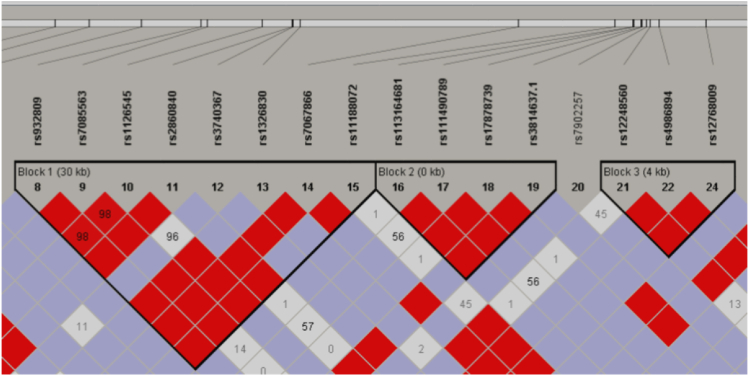
The first three LD blocks for CYP2C19 and CYP2C18 SNPs detected in our study.

**Figure 2:**
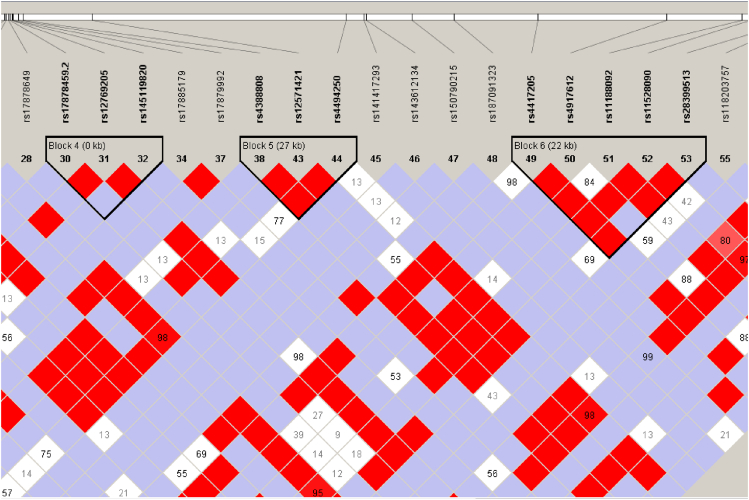
The second three LD blocks for CYP2C19 and CYP2C18 SNPs detected in our study.

**Figure 3:**
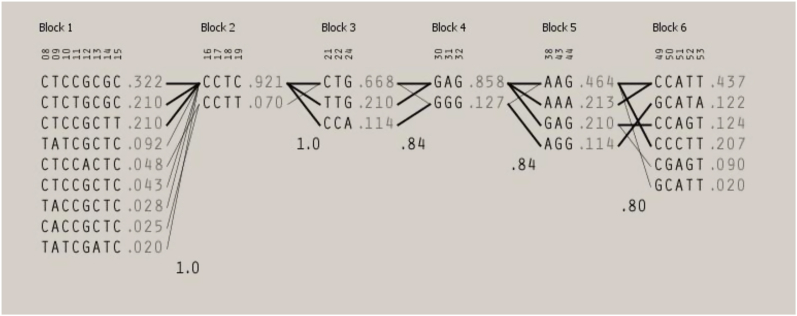
Haplotype structure across the six determined LD blocks.

We additionally identified six LD blocks with D′ ≥ 8 (Figure 1, Figure 2). The first was the largest block and the only block involving *CYP2C18* variants. It consisted of eight SNPs: six *CYP2C18* variants with inconclusive effects and two *CYP2C19* variants associated with ultra-metabolism. The strong linkage suggested that these specific *CYP2C18* variants were likely co-segregate with *CYP2C19* variants associated with elevated enzymatic activity. In this LD block, three haplotypes (CTCCGCGC, CTCTGCGC, and CTCCGCTT) were present among >20 % of the included participants (Figure 3).

LD block 2 was dominated by the CCTC haplotype, which involved rs3814637, a variant associated with a poor metabolizer phenotype. LD block 3 included variants with mixed effects on the CYP2C19 enzyme. The most frequently observed haplotypes (CTG and TTG) were associated with rapid-metabolizer and ultra-metabolizer phenotypes. Although less commonly detected than the two aforementioned haplotypes in LD block 3, the CCA haplotype, associated with the poor metabolizer phenotype, was observed among 11 % of participants (Figure 1, Figure 3).

The remaining LD blocks (blocks 4–6) consisted predominantly of variants associated with the inactive CYP2C19 enzyme. The minor allele frequencies among the identified haplotypes ranged between 11 % and 13 % (Figure 2, Figure 3).

## Discussion

This study examined 141 SNPs associated with the *CYP2C19* and *CYP2C18* genes and their allele and genotype frequencies in 374 Saudi individuals. The MAFs ranged from 0.13 % to 46.79 % for 53 SNPs. Several SNPs were not detected (n = 88) or had call rates <95 % (n = 5). Thirty-one (n = 31) SNPs were very common (MAF ≥5 %), including rs7067866 (47 %), rs7916649 (47 %), rs4917623 (43 %), rs7917985 (24 %), rs7915414 (22 %), rs11528090 (21 %), rs4494250 (21 %), rs2860840 (21 %), rs2901783 (22 %), and rs12268020 (22.0 %). The remaining 22 SNPs (MAF <5 %) were less common, including rs117140172 (2 %), rs1326830 (2 %), rs7902257 (2 %), rs941890 (3 %), rs11188059 (4 %), rs113164681 (1 %), rs150790215 (1 %), rs145119820 (1 %), rs60181876 (1 %), and rs17878459 (1 %).

Haplotype analysis identified two *CYP2C19* variants with MAF >10 % (rs4417205 and rs12768009) that were significantly associated with other well-known variants in a single haplotype. Additionally, four *CYP2C18* variants (rs932809, rs7085563, rs1126545, and rs1326830) were associated with one *CYP2C19* variant (rs7067866) previously suggested to be associated with the ultra-rapid metabolizer phenotype.^19^ Furthermore, the dominant haplotypes were associated with rapid-metabolizer phenotypes, followed by ultra-rapid metabolizer and poor-metabolizer phenotypes. Of note, in prior studies, CYP2C18 protein expression has been poorly detected in liver tissue but detected in other tissues, such as skin and lung, thus suggesting potential extrahepatic involvement of CYP2C18 in the metabolism of certain drugs. Moreover, some studies have associated certain *CYP2C18* variants with the metabolism of clopidogrel, warfarin, and phenytoin 20, 21∗, 22.

Several studies in the Middle East have examined the occurrence of common *CYP2C19* variants, including rs4244285 (*CYP2C19∗2*), rs4986893 (*CYP2C19∗3*), rs17878459 (*CYP2C19∗3*), rs28399504 (*CYP2C19∗4*), rs56337013 (*CYP2C19∗5*), rs2558184 (*CYP2C19∗6*), rs72558186 (*CYP2C19∗7*), rs41291556 (*CYP2C19∗8*), rs17884712 (*CYP2C19∗9*), rs6413438 (*CYP2C19∗10*), rs55640102 (*CYP2C19∗12*), rs17879685 (*CYP2C19∗13*), rs55752064 (*CYP2C19∗14*), rs17882687 (*CYP2C19∗15*), rs12248560 (*CYP2C19∗17*), and rs3758581 (*CYP2C19*∗1B). However, to our knowledge, no other gene variants associated with *CYP2C19* and its cognate *CYP2C18* have been assessed in Middle Eastern nations. Therefore, this study is the first, to our knowledge, to determine the presence of 141 genetic variants associated with the *CYP2C19* and *CYP2C18* genes in the Middle Eastern population.

According to the Single Nucleotide Polymorphism Database (dbSNP), several detected variants were associated with other populations (Table S3).^23^ For example, the MAF of rs7916649 (*CYP2C19*∗2B), a *CYP2C19* mutation associated with CYP2C19 loss-of-function, was similar to that in the global, European, and East Asian populations, but was less than that in African and Southeast Asian populations, and far higher than that in Latin American populations. This mutation was not measured in any prior study conducted in the Middle East.

In addition, in the Saudi population, the MAFs of rs12248560 and rs11188072, which are *CYP2C19* mutations associated with enhanced CYP2C19 function, were very close to the reference data for global, European, and African populations, but far exceeded those in East Asian, South Asian, and Latin American populations.

Furthermore, we detected the rs17878459 (*CYP2C19*∗3) variant among 0.7 % of study participants. This variant had not been detected or measured in previous studies in other Middle Eastern populations.^6^^,^^24^, ^25^, ^26^, ^27^, ^28^, ^29^, ^30^, ^31^, ^32^ Additionally, the variant rs12248560 (*CYP2C19*∗17) had a frequency of 21 %, a value lower than those in other Saudi studies but comparable to findings from Tunisian and Iraqi studies. Table S4 shows the *CYP2C19* star allele frequencies in our study compared with previous studies in Middle Eastern populations, including inhabitants of the Gulf region, Iraqis, Iranians, Israelis, Jordanians, Palestinians, Egyptians, Tunisians, and Lebanese.^6^^,^^24^, ^25^, ^26^, ^27^, ^28^, ^29^, ^30^, ^31^, ^32^

The previously poorly studied rs7916649 *CYP2C19* variant was a highly detected *CYP2C19* variant among the Saudi population. The rs7916649 variant is strongly associated with the *CYP2C19*∗2B and *CYP2C19*∗2C alleles and is probably associated with poor CYP2C19 enzymatic activity.^33^^,^^34^ According to Malki et al., the phenotype of needing valproic acid dose reduction is common among carriers of the rs7916649 variant (composite odds ratio of 1.95 (*p*-value <0.001) in carriers).^33^^,^^34^ Because Malki et al. used large samples from British biobanks linked to medical data, the study has provided relatively strong evidence that the rs7916649 variant is associated with poor valproic acid metabolism, thereby increasing exposure to adverse drug reactions.^33^^,^^34^

Moreover, in a 2015 study, 275 Chinese patients who carried the rs7916649 variant and used long-term clopidogrel had significantly greater risk of recurrent cardiac events, possibly because of treatment failure, than those with the homozygous *CYP2C19* wild-type genotype (*p*-value = 0.013). Despite the small sample size in that study, several studies have also suggested the rs7916649 variant as a possible reason for the poor metabolism of drugs reacting with the CYP2C19 enzyme.^35^

Patients diagnosed with coronary artery disease and neurological disorders frequently receive prescriptions for clopidogrel and valproic acid in KSA. Consequently, the likelihood of treatment failure and adverse outcomes might be predicted according to the identification of the rs7916649 mutation in >46 % of our study sample. Nonetheless, local pharmacogenomics investigations are necessary to Several studies have suggested that the rs4917623 variant is associated with poor CYP2C19 enzymatic activity. Yoshimoto et al.^36^ conducted a study that included 329 patients diagnosed with breast cancer who received tamoxifen treatment. The study revealed that patients carrying the rs4917623 TC and CC genotypes of the *CYP2C19* gene had enhanced anti-cancer efficacy for longer periods than the TT genotype. However, the difference was insignificant (*p*-value = 0.064). In addition, in a Chinese study of 105 patients who underwent kidney transplants and received tacrolimus as an immunosuppressant, the drug pharmacokinetics significantly differed between carriers of the rs4917623 variant and *CYP2C19* wild-type allele carriers.^37^ The rs4917623 variant was identified in 42.78 % of the Saudi population. Therefore, if local clinical trials confirm this association, Saudi individuals carrying the rs7916649 variant might require tacrolimus monitoring.

The rs2860840 variant is known to be associated with ultra-rapid metabolism of drugs biotransformed by CYP2C19 and CYP2C18 enzymes.^38^^,^^39^ Kee et al. have investigated the efficacy of omeprazole in the presence of multiple genetic variants in 67 individuals. The rs2860840 variant was associated with decreased concentrations of omeprazole, which might lead to treatment failure; carriers therefore may require higher doses than non-carriers.^40^

The rs12248560 variant, also known as *CYP2C19*∗17, has been extensively studied worldwide and confirmed to have ultra-rapid metabolism effects.^41^ This variant is significantly associated with in-hospital death among patients with acute coronary syndrome and non-valvular atrial fibrillation who receive clopidogrel with rivaroxaban. Additionally, carriers of the rs12248560 T allele have significantly higher rates of hyper-response to clopidogrel and higher rates of bleeding complications than carriers of the CC genotype.^41^ Carriers of the rs12248560 T allele have a significantly higher rate of hyper-response to prasugrel and a higher rate of bleeding complications than carriers of the CC genotype.^42^ Furthermore, carriers of the rs12248560 allele might have greater imipramine metabolism than carriers of two normal alleles. Yet, this effect of the rs12248560 allele does not alter the clinical outcomes due to the activity of imipramine's active metabolite, which is desipramine.^43^ Nonetheless, carriers of the rs12248560 allele might have greater citalopram metabolism than carriers of two normal alleles. However, conflicting evidence has been reported.^44^ Furthermore, carriers of the rs12248560 allele might exhibit greater voriconazole metabolism than carriers of two normal alleles. However, because of uncertainty in the findings, comprehensive local trials are necessary.^45^

Several studies have associated the rs11188072 variant with ultra-rapid metabolism of drugs biotransformed by the CYP2C19 enzyme. Carriers of the rs11188072 T allele have an elevated risk of omeprazole treatment failure.^46^ Furthermore, carriers of the rs11188072 T allele might have diminished risk of cardiovascular events, because of enhanced clopidogrel response and antiplatelet action. However, a meta-analysis by Bauer et al. has revealed insignificant differences in clopidogrel efficacy among carriers of several rs11188072 genotypes.^47^^,^^48^ In addition, mephenytoin blood concentrations are extremely low among carriers of the rs11188072 TT genotype, in contrast to the wild-type genotype.^46^

The rs12769205 variant, also known as *CYP2C19*∗2E or *CYP2C19*∗2J, is significantly associated with diminished metabolism of drugs biotransformed by the CYP2C19 enzyme. Pharmacokinetic studies have indicated that tricyclic antidepressants, including imipramine, clomipramine, trimipramine, amitriptyline, and doxepin, are poorly metabolized, and their blood levels are elevated, among carriers of the rs12769205 mutant allele G (GG or AG). The Clinical Pharmacogenetics Implementation Consortium dosing guideline update recommends considering a 50 % dose reduction in carriers of the rs12769205 mutant allele G.^49^ In addition, carriers of the rs12769205 mutant allele G might show a better response to proton pump inhibitors, including lansoprazole, dexlansoprazole, pantoprazole, rabeprazole, esomeprazole, and omeprazole, than carriers of two normal alleles. Hence, a 50 % dose reduction might be considered for carriers of the rs12769205 mutant allele G if proton pump inhibitors are to be used for long periods (>3 months); however, local studies should be conducted to validate this finding.^50^

The rs12571421 variant is likely to be associated with poor metabolism of drugs biotransformed by the CYP2C19 enzyme. Carriers of the minor allele of the rs12571421 variant have higher risk of clopidogrel resistance than wild-type allele carriers.^51^ In addition, patients taking either citalopram or escitalopram who carry the minor allele of the rs12571421 variant might have better antidepressant efficacy, but a higher risk of adverse events due to poor metabolism, than wild-type allele carriers.^52^ Furthermore, HIV-infected patients taking etravirine who carry the minor allele of the rs12571421 variant might have better anti-HIV efficacy, but a higher risk of adverse events due to poor metabolism, than wild-type allele carriers.^53^^,^^54^ Moreover, women who carry the minor allele of the rs12571421 variant might have lower norendoxifen (an aromatase inhibitor and active metabolite of tamoxifen) concentrations, due to poor tamoxifen metabolism, than wild-type allele carriers.^55^ No published studies have correlated any disease with the rs12571421 variant.

This study has several limitations, notably the omission of the rs4244285 (*CYP2C19*∗2) variant, whose influence on the efficiency of common medications is well established among carriers. Because the main objective of this study was to determine the frequencies of numerous unmeasured *CYP2C19* and *CYP2C18* genetic variations in KSA, we focused on examining previously unmeasured or minimally measured variants. Hence, rs4244285 (*CYP2C19*∗2) was omitted, because its frequency in KSA and globally has been documented. We acknowledge that this omission affected the assessment of complete haplotype analysis and limited the interpretation of associations between the observed *CYP2C19* and *CYP2C18* variants, particularly those with unconfirmed implications. Nonetheless, the key *CYP2C19*∗2 variants are the two splicing variants rs4244285 and rs12769205, both of which are known to be closely related, to map to the same haplotype, and to be associated with CYP2C19 loss of function.^56^^,^^57^ Hence, the effects of this omission were partially mitigated by the inclusion of rs12769205.

Furthermore, several variants were recognized as rare and lacked sufficient statistical power for association analyses, thus restricting interpretability. Additionally, the therapeutic importance of numerous identified polymorphisms affecting the CYP2C19 and CYP2C18 enzymes remains either uncertain or unverified, because in silico functional predictions, including SIFT, CADD, and DANN, have not been experimentally validated. Therefore, to establish an association between a certain genetic variant and particular medications, in vitro validation for high-impact variants should be followed by in vivo studies with a sufficient sample size.

## Conclusion

This study investigated the presence of many genetic variants associated with the *CYP2C19* and *CYP2C18* genes that were previously unreported in the Middle Eastern population, including the Saudi population. The most common (MAF >10 %) and important detected genetic polymorphisms were rs12571421, rs12248560, rs7916649, rs12769205, rs11188072, rs2860840, and rs4917623, which are likely to affect the metabolism of essential drugs for treating severe cases of epilepsy, fungal infections, cardiovascular diseases, kidney failure, and psychiatric diseases. Nonetheless, further pharmacogenomics investigations will be necessary to validate the effects of these variants and haplotypes through high-quality in vitro, in vivo, and clinical association studies.

### Future directions

The current clinical guidelines for pharmacogenomics and medical genetics, such as those from the Clinical Pharmacogenetics Implementation Consortium and the American College of Medical Genetics and Genomics, have relied on studies conducted among non-Middle Eastern populations. Consequently, several recommendations regarding *CYP2C19* and *CYP2C18* genetic variants might not precisely reflect the Middle Eastern genetic profile. Our findings establish an important local baseline and suggest that locally adapted guidelines will be essential to focus on the observed haplotypes in future research. By integrating the current outcomes associated with variants with established associations, the healthcare systems of KSA and other Middle Eastern countries could optimize prophylactic measures, drug selection, and dosing for CYP2C19 and CYP2C18 substrates, thereby moving toward effective application of personalized medicine.

## Source of funding

The authors extend their appreciation to the Deanship of Scientific Research, Vice Presidency for Graduate Studies and Scientific Research, King Faisal University, Saudi Arabia (Grant Number: KFU261172).

## Ethical approval

The research protocol for this study was approved by the Research Ethics Committee of King Fahad Hospital (IRB log number 27/A-29-2020) and Research Ethics Committee of King Faisal University (IRB log number KFU-REC/2021-03-09). Before participants’ involvement in the study, the researchers obtained written informed consent. Participants' confidentiality and anonymity were ensured by assigning each participant a unique code number solely for analysis purposes. Participation in the present study was entirely voluntary.

## Authors contributions

All the authors made substantial contributions to the conception of the study.

AK: conceptualization, methodology, investigation, data curation, formal analysis, writing—original draft; AH and YM: investigation, resources, formal analysis, writing—original draft; MK: resources, validation, writing—review and editing, supervision; AT: conceptualization, methodology, data curation, formal analysis, writing—review and editing, supervision.

All authors have critically reviewed and approved the final draft and are responsible for the content and similarity index of the manuscript.

## Data availability

The authors confirm that the data supporting the findings of this study are available within the article and its supplementary materials.

## Conflicts of interest

The authors declare no conflicts of interest.

## References

[1] Murayama N., Imai N., Nakane T. (2007). Roles of CYP3A4 and CYP2C19 in methyl hydroxylated and N-oxidized metabolite formation from voriconazole, a new anti-fungal agent, in human liver microsomes. Biochem Pharmacol.

[2] Goldstein J.A. (2001). Clinical relevance of genetic polymorphisms in the human CYP2C subfamily. Br J Clin Pharmacol.

[3] Strawn J.R., Poweleit E.A., Ramsey L.B. (2019). CYP2C19-Guided escitalopram and sertraline dosing in pediatric patients: a pharmacokinetic modeling study. J Child Adolesc Psychopharmacol.

[4] De Vos A., Van Der Weide J., Loovers H.M. (2011). Association between CYP2C19∗17 and metabolism of amitriptyline, citalopram and clomipramine in Dutch hospitalized patients. Pharmacogenomics J.

[5] Lee S.J. (2013). Clinical application of CYP2C19 pharmacogenetics toward more personalized medicine. Front Genet.

[6] Al-Jenoobi F.I., Alkharfy K.M., Alghamdi A.M. (2013). CYP2C19 genetic polymorphism in Saudi Arabians. Basic Clin Pharmacol Toxicol.

[7] Zubiaur P., Gaedigk A. (2022). CYP2C18: the orphan in the CYP2C family. Pharmacogenomics.

[8] Liu J., Nie X.-Y., Zhang Y. (2015). CYP2C19∗2 and other allelic variants affecting platelet response to clopidogrel tested by thrombelastography in patients with acute coronary syndrome. Chin Med J (Engl)..

[9] Desta Z., Zhao X., Shin J.G. (2002). Clinical significance of the cytochrome P450 2C19 genetic polymorphism. Clin Pharmacokinet.

[10] Cacabelos R., Cacabelos P., Torrellas C. (2014).

[11] Alhazzani A.A., Munisamy M., Karunakaran G. (2017). Pharmacogenetics of CYP2C19 genetic polymorphism on clopidogrel response in patients with ischemic stroke from Saudi Arabia. Neurosciences (Riyadh).

[12] Alkattan A., Alsalameen E. (2021). Polymorphisms of genes related to phase-I metabolic enzymes affecting the clinical efficacy and safety of clopidogrel treatment. Expert Opin Drug Metab Toxicol.

[13] Saeed L.H., Mayet A.Y. (2013). Genotype-phenotype analysis of CYP2C19 in healthy Saudi individuals and its potential clinical implication in drug therapy. Int J Med Sci.

[14] Alqahtany A. (2023). GIS-based assessment of land use for predicting increase in settlements in Al Ahsa metropolitan area, Saudi Arabia for the year 2032. Alexandria Eng J.

[15] Zhou Y., Mkrtchian S., Kumondai M. (2019). An optimized prediction framework to assess the functional impact of pharmacogenetic variants. Pharmacogenomics J.

[16] Konjhodzic R., Salihefendic L., Mulahuseinovic N. (2024). Novel intronic heterozygous mutation in TSC2 gene in pediatric patient with Tuberous sclerosis complex. Acta Inf Med.

[17] Dong S.S., He W.M., Ji J.J. (2021). LDBlockShow: a fast and convenient tool for visualizing linkage disequilibrium and haplotype blocks based on variant call format files. Brief Bioinform.

[18] Suzuki H., Ota M., Meguro A. (2012). Genetic characterization and susceptibility for sarcoidosis in Japanese patients: risk factors of BTNL2 gene polymorphisms and HLA class II alleles. Investig Ophthalmol Vis Sci.

[19] Janha R.E., Sisay-Joof F., Hamid-Adiamoh M. (2009). Effects of genetic variation at the CYP2C19/CYP2C9 locus on pharmacokinetics of chlorcycloguanil in adult Gambians. Pharmacogenomics.

[20] Chung W.H., Hung S.I., inventors; National Yang Ming University NYMU, Chang Gung Medical Foundation Chang Gung Memorial Hospital at Keelung, assignee (2021). Risk assessment for phenytoin-induced adverse drug reactions. United States patent US.

[bib21] Backman J.D., O'Connell J.R., Tanner K. (2017). Genome-wide analysis of clopidogrel active metabolite levels identifies novel variants that influence antiplatelet response. Pharmacogenet Genom.

[22] Asiimwe I.G., Blockman M., Cohen K. (2022). A genome-wide association study of plasma concentrations of warfarin enantiomers and metabolites in sub-saharan Black-African patients. Front Pharmacol.

[23] Phan L., Zhang H., Wang Q. (2025). The evolution of dbSNP: 25 years of impact in genomic research. Nucleic Acids Res.

[24] Mirghani R.A., Chowdhary G., Elghazali G. (2011). Distribution of the major cytochrome P450 (CYP) 2C9 genetic variants in a Saudi population. Basic Clin Pharmacol Toxicol.

[25] Abdelhedi R., Bouayed N.A., Alfadhli S. (2015). Characterization of drug-metabolizing enzymes CYP2C9, CYP2C19 polymorphisms in Tunisian, Kuwaiti and Bahraini populations. J Genet.

[26] Sahib H.A., Mohammed B., Abdul-Majid B.A. (2015). Genetic polymorphism of CYP2C19 in a sample of Iraqi population. Int J Pharm Biol Sci.

[27] Khalil B.M., Shahin M.H., Solayman M.H. (2016). Genetic and nongenetic factors affecting clopidogrel response in the Egyptian population. Clin Transl Sci.

[28] Sviri S., Shpizen S., Leitersdorf E. (1999). Phenotypic-genotypic analysis of CYP2C19 in the Jewish Israeli population. Clin Pharmacol Ther.

[29] Ayesh B.M., Al-Astal I.R., Yassin M.M. (2019). The clinical effects of CYP2C19 ∗2 allele frequency on Palestinian patients receiving clopidogrel after percutaneous coronary intervention. Int J Clin Pharm.

[30] Ahmad M., Navarro-Quiroz E., García Moreno A.M. (2018). Analysis of gene polymorphism CYP2C19 in the Lebanese population who reside in Colombia. Global J Health Sci.

[31] Rjoub M., Saleh A., Hakooz N. (2018). Allelic frequency of PON1 Q192R, CYP2C19∗ 2 and CYP2C19∗ 17 among Jordanian patients taking clopidogrel. Trop J Pharmaceut Res.

[32] Payan M., Tajik N., Rouini M.R. (2015). Genotype and allele frequency of CYP2C19∗ 17 in a healthy Iranian population. Med J Islam Repub Iran.

[33] Malki M.A. (2021). https://discovery.dundee.ac.uk/ws/portalfiles/portal/57075052/1.2_Final_PhD_Thesis_DGIs_and_DDGIs_for_commonly_used_drugs.pdf.

[34] Malki M.A., Dawed A.Y., Haywood C. (2021). Utilizing large electronic medical record data sets to identify novel drug-gne interactions for commonly used drugs. Clin Pharmacol Ther.

[bib35] Liu M., Xiao F., Wang X. (2015). 基因多态性, 药物联用和临床因素对氯吡格雷疗效的影响及相关的基因型分布 [effects of gene polymorphism, drug combination and clinical factors on the efficacy of clopidogrel and related genotype distribution]. Chin J Clin Pharmacol Ther.

[bib36] Yoshimoto N., Naito A., Kawaguchi N. (2019). The CYP2C19 rs4917623 single nucleotide polymorphism to predict tamoxifen efficacy in estrogen receptor-positive breast cancer patients. J Clin Oncol.

[bib37] Rahamimov R., Chagnac A., Mor E. (2018). Long term exposure to high variability of tacrolimus trough levels and exposure to sub therapeutic drug levels predicts worse survival after kidney transplantation. Transplantation.

[38] Bråten L.S., Haslemo T., Jukic M.M. (2021). A Novel CYP2C-Haplotype associated with ultrarapid metabolism of escitalopram. Clin Pharmacol Ther.

[39] Bråten L.S., Ingelman-Sundberg M., Jukic M.M. (2022). Impact of the novel CYP2C:TG haplotype and CYP2B6 variants on sertraline exposure in a large patient population. Clin Transl Sci.

[40] Kee P.S., Maggo S.D.S., Kennedy M.A. (2022). Omeprazole treatment failure in gastroesophageal reflux disease and genetic variation at the CYP2C locus. Front Genet.

[41] Sychev D.A., Baturina O.A., Mirzaev K.B. (2020). CYP2C19∗17 may increase the risk of death among patients with an acute coronary syndrome and non-valvular atrial fibrillation who receive clopidogrel and rivaroxaban. Pharmgenomics Pers Med.

[42] Cuisset T., Loosveld M., Morange P.E. (2012). CYP2C19∗2 and ∗17 alleles have a significant impact on platelet response and bleeding risk in patients treated with prasugrel after acute coronary syndrome. JACC Cardiovasc Interv.

[43] Hicks J.K., Swen J.J., Thorn C.F. (2013). Clinical Pharmacogenetics Implementation consortium guideline for CYP2D6 and CYP2C19 genotypes and dosing of tricyclic antidepressants. Clin Pharmacol Ther.

[44] Hicks J.K., Bishop J.R., Sangkuhl K. (2015). Clinical pharmacogenetics implementation consortium (CPIC) guideline for CYP2D6 and CYP2C19 genotypes and dosing of selective serotonin reuptake inhibitors. Clin Pharmacol Ther.

[45] Moriyama B., Obeng A.O., Barbarino J. (2017). Clinical pharmacogenetics implementation consortium (CPIC) guidelines for CYP2C19 and voriconazole therapy. Clin Pharmacol Ther.

[46] Sim S.C., Risinger C., Dahl M.L. (2006). A common novel CYP2C19 gene variant causes ultrarapid drug metabolism relevant for the drug response to proton pump inhibitors and antidepressants. Clin Pharmacol Ther.

[47] Yasmina A., De Boer A., Klungel O.H. (2014). Pharmacogenomics of oral antiplatelet drugs. Pharmacogenomics.

[48] Bauer T., Bouman H.J., van Werkum J.W. (2011). Impact of CYP2C19 variant genotypes on clinical efficacy of antiplatelet treatment with clopidogrel: systematic review and meta-analysis. BMJ.

[bib49] Hicks J.K., Sangkuhl K., Swen J.J. (2017). Clinical pharmacogenetics implementation consortium guideline (CPIC) for CYP2D6 and CYP2C19 genotypes and dosing of tricyclic antidepressants: 2016 update. Clin Pharmacol Ther.

[50] Willcocks I.R., Legge S.E., Nalmpanti M. (2021). Clozapine metabolism is associated with absolute neutrophil count in individuals with treatment-resistant schizophrenia. Front Pharmacol.

[51] Giri A.K., Khan N.M., Basu A. (2014). Pharmacogenetic landscape of clopidogrel in north Indians suggest distinct interpopulation differences in allele frequencies. Pharmacogenomics.

[52] Ji Y., Schaid D.J., Desta Z. (2014). Citalopram and escitalopram plasma drug and metabolite concentrations: genome-wide associations. Br J Clin Pharmacol.

[53] Di Francia R., Fierro C., Di Paolo M. (2015). Selected pharmacogenetic panel test for toxicity prevention of drug-drug interactions between highly active antiretroviral therapy (HAART) and antiblastic chemotherapy. WCRJ.

[54] McLaren P.J., Telenti A. (2016).

[55] Lim J.S., Sutiman N., Muerdter T.E. (2016). Association of CYP2C19∗2 and associated haplotypes with lower norendoxifen concentrations in tamoxifen-treated Asian breast cancer patients. Br J Clin Pharmacol.

[bib56] Díaz-Ordóñez L., Ramírez-Montaño D., Candelo E. (2021). Evaluation of CYP2C19 gene polymorphisms in patients with acid peptic disorders treated with esomeprazole. Pharmgenomics Pers Med.

[57] Pharmacogene variation consortium. CYP2C19. Accessed 2025 May 11. Available at https://www.pharmvar.org/gene/cyp2c19/.

