# Supplementary material for: Genetic variants of CYP2C19 and CYP2C18 and their clinical implications in the Saudi population
Source: J Taibah Univ Med Sci. 2026 Mar 20;21(2):307–21. doi: 10.1016/j.jtumed.2026.03.003 (PMC13018941; doi:10.1016/j.jtumed.2026.03.003)
Supplement: Multimedia component 1 [file mmc1.docx]

**Table S1 *CYP2C19* and *CYP2C18* measured variants (N=146) with their chromosomal positions, Gentrain score, Call frequency, number of calls, and no calls.**

| n | Variant name | Chr | Position | ChiTest100 | GenTrain Score | AA Freq | AB Freq | BB Freq | Call Freq | MAF | SNP | # Calls | # no calls |
| --- | --- | --- | --- | --- | --- | --- | --- | --- | --- | --- | --- | --- | --- |
| 1 | rs117111102 | 10 | 96447920 | 1 | 0.8916 | 0 | 0 | 1 | 1 | 0 | [T/C] | 374 | 0 |
| 2 | rs150562893 | 10 | 96448186 | 1 | 0.8818 | 1 | 0 | 0 | 1 | 0 | [T/C] | 374 | 0 |
| 3 | rs183718557 | 10 | 96448585 | 1 | 0.8172 | 0 | 0 | 1 | 1 | 0 | [T/C] | 374 | 0 |
| 4 | rs201633987 | 10 | 96451136 | 1 | 0.6804 | 1 | 0 | 0 | 1 | 0 | [A/G] | 374 | 0 |
| 5 | rs2901783 | 10 | 96453099 | 0.5544 | 0.8848 | 0.6176 | 0.3235 | 0.0588 | 1 | 0.2206 | [T/C] | 374 | 0 |
| 6 | rs11188056 | 10 | 96454700 | 1 | 0.8646 | 0 | 0 | 1 | 1 | 0 | [A/G] | 374 | 0 |
| 7 | rs141271146 | 10 | 96454748 | 1 | 0.9096 | 0 | 0 | 1 | 1 | 0 | [T/C] | 374 | 0 |
| 8 | rs115269792 | 10 | 96454768 | 1 | 0.8901 | 0 | 0 | 1 | 1 | 0 | [C/G] | 374 | 0 |
| 9 | rs148902102 | 10 | 96461071 | 1 | 0.8508 | 1 | 0 | 0 | 1 | 0 | [A/C] | 374 | 0 |
| 10 | rs7900135 | 10 | 96465616 | 0.2123 | 0.8594 | 0.754 | 0.2112 | 0.0348 | 1 | 0.1404 | [T/C] | 374 | 0 |
| 11 | rs2296681 | 10 | 96466592 | 1 | 0.8709 | 1 | 0 | 0 | 1 | 0 | [A/G] | 374 | 0 |
| 12 | rs184571563 | 10 | 96466679 | 1 | 0.8909 | 0 | 0 | 1 | 1 | 0 | [T/C] | 374 | 0 |
| 13 | rs117140172 | 10 | 96467850 | 0.8333 | 0.8196 | 0.0027 | 0.0348 | 0.9626 | 1 | 0.0201 | [A/G] | 374 | 0 |
| 14 | rs560806209 | 10 | 96468870 | 1 | 0.8949 | 0 | 0 | 1 | 1 | 0 | [A/G] | 374 | 0 |
| 15 | rs11188059 | 10 | 96468899 | 0.6584 | 0.8684 | 0 | 0.0909 | 0.9091 | 1 | 0.0455 | [A/G] | 374 | 0 |
| 16 | rs7917985 | 10 | 96469632 | 0.4844 | 0.7914 | 0.0428 | 0.385 | 0.5722 | 1 | 0.2353 | [A/G] | 374 | 0 |
| 17 | rs186024974 | 10 | 96475841 | 1 | 0.7026 | 1 | 0 | 0 | 1 | 0 | [A/G] | 374 | 0 |
| 18 | rs150579865 | 10 | 96477700 | 1 | 0.897 | 0 | 0.0107 | 0.9893 | 1 | 0.0053 | [A/C] | 374 | 0 |
| 19 | rs139191072 | 10 | 96479332 | 1 | 0.858 | 0 | 0 | 1 | 0.9947 | 0 | [T/G] | 372 | 2 |
| 20 | rs149917524 | 10 | 96479475 | 1 | 0.6149 | 0 | 0 | 1 | 0.9973 | 0 | [T/C] | 373 | 1 |
| 21 | rs60181876 | 10 | 96480229 | 1 | 0.9012 | 0 | 0.0134 | 0.9866 | 1 | 0.0067 | [T/C] | 374 | 0 |
| 22 | rs59636573 | 10 | 96484129 | 1 | 0.8331 | 0 | 0.0027 | 0.9973 | 1 | 0.0013 | [T/G] | 374 | 0 |
| 23 | rs41286880 | 10 | 96484145 | 1 | 0.8745 | 0 | 0 | 1 | 0.992 | 0 | [T/C] | 371 | 3 |
| 24 | rs41286882 | 10 | 96484153 | 1 | 0.8004 | 1 | 0 | 0 | 1 | 0 | [T/C] | 374 | 0 |
| 25 | rs932809 | 10 | 96488469 | 0.2123 | 0.8806 | 0.0348 | 0.2112 | 0.754 | 1 | 0.1404 | [T/C] | 374 | 0 |
| 26 | rs7085563 | 10 | 96488997 | 0.3613 | 0.699 | 0.0401 | 0.2513 | 0.7086 | 1 | 0.1658 | [T/A] | 374 | 0 |
| 27 | rs1126545 | 10 | 96493058 | 0.4004 | 0.8176 | 0.0214 | 0.1845 | 0.7941 | 1 | 0.1136 | [T/C] | 374 | 0 |
| 28 | rs79500998 | 10 | 96495052 | 1 | 0.8815 | 0 | 0 | 1 | 1 | 0 | [T/C] | 374 | 0 |
| 29 | rs41286884 | 10 | 96495161 | 1 | 0.8181 | 0 | 0 | 1 | 1 | 0 | [A/G] | 374 | 0 |
| 30 | rs2860840 | 10 | 96495232 | 0.9122 | 0.7962 | 0.0428 | 0.3369 | 0.6203 | 1 | 0.2112 | [T/C] | 374 | 0 |
| 31 | rs3740367 | 10 | 96495266 | 0.9412 | 0.8559 | 0.0027 | 0.0909 | 0.9064 | 1 | 0.0481 | [T/C] | 374 | 0 |
| 32 | rs41291552 | 10 | 96495410 | 1 | 0.8788 | 0 | 0 | 1 | 1 | 0 | [A/G] | 374 | 0 |
| 33 | rs1326830 | 10 | 96495793 | 0.9703 | 0.8809 | 0 | 0.0401 | 0.9599 | 1 | 0.0201 | [A/C] | 374 | 0 |
| 34 | rs7067866 | 10 | 96511926 | 0.5998 | 0.8403 | 0.2059 | 0.5241 | 0.2701 | 1 | 0.4679 | [A/C] | 374 | 0 |
| 35 | rs11188072 | 10 | 96519061 | 0.3773 | 0.8272 | 0.0294 | 0.361 | 0.6096 | 1 | 0.2099 | [A/G] | 374 | 0 |
| 36 | rs113164681 | 10 | 96520433 | 0.9996 | 0.8759 | 0 | 0.0187 | 0.9813 | 1 | 0.0094 | [T/C] | 374 | 0 |
| 37 | rs111490789 | 10 | 96520443 | 1 | 0.5497 | 0 | 0.0081 | 0.9919 | 0.9893 | 0.0041 | [T/G] | 370 | 4 |
| 38 | rs17878739 | 10 | 96521024 | 0.9996 | 0.5497 | 0.9813 | 0.0187 | 0 | 1 | 0.0094 | [A/G] | 374 | 0 |
| 39 | rs3814637 | 10 | 96521045 | 0.9029 | 0.9019 | 0.0054 | 0.1475 | 0.8472 | 0.9973 | 0.0791 | [A/G] | 373 | 1 |
| 40 | rs7902257 | 10 | 96521422 | 0.7758 | 0.8933 | 0.0027 | 0.0401 | 0.9572 | 1 | 0.0227 | [A/G] | 374 | 0 |
| 41 | rs12248560 | 10 | 96521657 | 0.3773 | 0.8599 | 0.0294 | 0.361 | 0.6096 | 1 | 0.2099 | [T/C] | 374 | 0 |
| 42 | rs4986894 | 10 | 96522365 | 0.4004 | 0.8738 | 0.7941 | 0.1845 | 0.0214 | 1 | 0.1136 | [T/C] | 374 | 0 |
| 43 | rs367543001 | 10 | 96522450 | 0 | 0.4008 | 0 | 0 | 0 | 0 | 0 | [A/G] | 0 | 374 |
| 44 | rs55752064 | 10 | 96522512 | 1 | 0.8942 | 1 | 0 | 0 | 1 | 0 | [A/G] | 374 | 0 |
| 45 | rs1564656981 | 10 | 96522545 | 1 | 0.8172 | 1 | 0 | 0 | 0.992 | 0 | [T/A] | 371 | 3 |
| 46 | rs1564657013 | 10 | 96522613 | 1 | 0.8831 | 1 | 0 | 0 | 1 | 0 | [A/G] | 374 | 0 |
| 47 | rs572853437 | 10 | 96522626 | 1 | 0.9069 | 0 | 0 | 1 | 1 | 0 | [T/G] | 374 | 0 |
| 48 | rs148954322 | 10 | 96523959 | 1 | 0.5383 | 0 | 0 | 1 | 0.9947 | 0 | [T/G] | 372 | 2 |
| 49 | rs12768009 | 10 | 96525865 | 0.2642 | 0.7821 | 0.0267 | 0.1898 | 0.7834 | 1 | 0.1217 | [T/C] | 374 | 0 |
| 50 | rs116992754 | 10 | 96528531 | 1 | 0.6986 | 0 | 0 | 1 | 0.9866 | 0 | [T/G] | 369 | 5 |
| 51 | rs199904246 | 10 | 96530760 | 1 | 0.6532 | 0 | 0 | 1 | 1 | 0 | [T/C] | 374 | 0 |
| 52 | rs545579039 | 10 | 96530836 | 1 | 0.5902 | 1 | 0 | 0 | 1 | 0 | [A/G] | 374 | 0 |
| 53 | rs185348086 | 10 | 96530908 | 1 | 0.7135 | 0 | 0 | 1 | 1 | 0 | [T/C] | 374 | 0 |
| 54 | rs189423009 | 10 | 96532728 | 0 | 0.4587 | 0 | 0 | 0 | 0 | 0 | [T/C] | 0 | 374 |
| 55 | rs187921622 | 10 | 96533546 | 1 | 0.66 | 0 | 0 | 1 | 1 | 0 | [T/G] | 374 | 0 |
| 56 | rs17884832 | 10 | 96534475 | 0.9372 | 0.8479 | 0.8663 | 0.1283 | 0.0053 | 1 | 0.0695 | [A/C] | 374 | 0 |
| 57 | rs7916649 | 10 | 96534584 | 0.6351 | 0.8395 | 0.2059 | 0.5214 | 0.2727 | 1 | 0.4666 | [A/G] | 374 | 0 |
| 58 | rs17878649 | 10 | 96534768 | 1 | 0.8893 | 0 | 0.0053 | 0.9947 | 1 | 0.0027 | [T/C] | 374 | 0 |
| 59 | rs145328984 | 10 | 96534863 | 1 | 0.8004 | 0 | 0 | 1 | 1 | 0 | [T/C] | 374 | 0 |
| 60 | rs28399505 | 10 | 96534867 | 1 | 0.7914 | 1 | 0 | 0 | 1 | 0 | [T/C] | 374 | 0 |
| 61 | rs1564660997 | 10 | 96534878 | 1 | 0.8004 | 0 | 0 | 1 | 1 | 0 | [T/C] | 374 | 0 |
| 62 | rs118203756 | 10 | 96534917 | 1 | 0.8234 | 0 | 0 | 1 | 1 | 0 | [G/C] | 374 | 0 |
| 63 | rs17878459 | 10 | 96534922 | 1 | 0.8294 | 0 | 0.0134 | 0.9866 | 1 | 0.0067 | [G/C] | 374 | 0 |
| 64 | rs1288601658 | 10 | 96534942 | 1 | 0.8275 | 1 | 0 | 0 | 1 | 0 | [A/G] | 374 | 0 |
| 65 | rs545642100 | 10 | 96534963 | 1 | 0.8115 | 0 | 0 | 1 | 1 | 0 | [G/C] | 374 | 0 |
| 66 | rs12769205 | 10 | 96535124 | 0.2277 | 0.4355 | 0.7513 | 0.2139 | 0.0348 | 1 | 0.1417 | [T/C] | 374 | 0 |
| 67 | rs145119820 | 10 | 96535152 | 0.9999 | 0.7174 | 0 | 0.016 | 0.984 | 1 | 0.008 | [T/C] | 374 | 0 |
| 68 | rs41291556 | 10 | 96535173 | 1 | 0.9074 | 1 | 0 | 0 | 1 | 0 | [T/C] | 374 | 0 |
| 69 | rs17885179 | 10 | 96535180 | 1 | 0.8586 | 0.992 | 0.008 | 0 | 1 | 0.004 | [A/C] | 374 | 0 |
| 70 | rs72552267 | 10 | 96535210 | 1 | 0.6916 | 0 | 0 | 1 | 1 | 0 | [A/G] | 374 | 0 |
| 71 | rs17884712 | 10 | 96535246 | 1 | 0.8136 | 1 | 0 | 0 | 1 | 0 | [C/G] | 374 | 0 |
| 72 | rs58973490 | 10 | 96535264 | 1 | 0.5548 | 0 | 0 | 1 | 0.9759 | 0 | [T/C] | 365 | 9 |
| 73 | rs17879992 | 10 | 96535628 | 0.9915 | 0.4759 | 0.8579 | 0.1367 | 0.0054 | 0.9973 | 0.0737 | [T/C] | 373 | 1 |
| 74 | rs4388808 | 10 | 96536056 | 0.9388 | 0.7986 | 0.623 | 0.3342 | 0.0428 | 1 | 0.2099 | [T/C] | 374 | 0 |
| 75 | rs140278421 | 10 | 96540331 | 1 | 0.9163 | 1 | 0 | 0 | 1 | 0 | [C/G] | 374 | 0 |
| 76 | rs370803989 | 10 | 96540336 | 1 | 0.5162 | 0 | 0 | 1 | 0.9786 | 0 | [A/G] | 366 | 8 |
| 77 | rs4986893 | 10 | 96540410 | 1 | 0.8872 | 0 | 0 | 1 | 1 | 0 | [A/G] | 374 | 0 |
| 78 | rs200936950 | 10 | 96540422 | 1 | 0.8795 | 0 | 0 | 1 | 1 | 0 | [A/G] | 374 | 0 |
| 79 | rs59162501 | 10 | 96541563 | 1 | 0.8611 | 1 | 0 | 0 | 1 | 0 | [T/C] | 374 | 0 |
| 80 | rs6413438 | 10 | 96541615 | 1 | 0.9018 | 0 | 0 | 1 | 1 | 0 | [T/C] | 374 | 0 |
| 81 | rs72558185 | 10 | 96541654 | 1 | 0.8911 | 1 | 0 | 0 | 1 | 0 | [D/I] | 374 | 0 |
| 82 | rs375781227 | 10 | 96541701 | 1 | 0.9034 | 0 | 0 | 1 | 1 | 0 | [T/C] | 374 | 0 |
| 83 | rs577255883 | 10 | 96541719 | 1 | 0.8296 | 0 | 0 | 1 | 1 | 0 | [A/G] | 374 | 0 |
| 84 | rs778258371 | 10 | 96541748 | 1 | 0.8949 | 0 | 0 | 1 | 1 | 0 | [T/C] | 374 | 0 |
| 85 | rs72558186 | 10 | 96541756 | 1 | 0.8999 | 1 | 0 | 0 | 1 | 0 | [T/A] | 374 | 0 |
| 86 | rs12571421 | 10 | 96541982 | 0.4004 | 0.9124 | 0.7941 | 0.1845 | 0.0214 | 1 | 0.1136 | [T/C] | 374 | 0 |
| 87 | rs143138959 | 10 | 96542340 | 1 | 0.8651 | 0 | 0 | 1 | 1 | 0 | [C/G] | 374 | 0 |
| 88 | rs373408094 | 10 | 96548860 | 1 | 0.6379 | 1 | 0 | 0 | 1 | 0 | [T/C] | 374 | 0 |
| 89 | rs139840199 | 10 | 96550349 | 1 | 0.6191 | 1 | 0 | 0 | 1 | 0 | [A/G] | 374 | 0 |
| 90 | rs528218818 | 10 | 96555606 | 1 | 0.7016 | 1 | 0 | 0 | 1 | 0 | [T/G] | 374 | 0 |
| 91 | rs192289355 | 10 | 96556364 | 1 | 0.6314 | 1 | 0 | 0 | 1 | 0 | [A/G] | 374 | 0 |
| 92 | rs551320091 | 10 | 96556784 | 1 | 0.5871 | 0 | 0 | 1 | 1 | 0 | [T/C] | 374 | 0 |
| 93 | rs140554854 | 10 | 96561715 | 1 | 0.7771 | 0 | 0 | 1 | 1 | 0 | [A/C] | 374 | 0 |
| 94 | rs555752933 | 10 | 96561872 | 1 | 0.6461 | 1 | 0 | 0 | 1 | 0 | [A/G] | 374 | 0 |
| 95 | rs575616160 | 10 | 96561874 | 1 | 0.5291 | 0 | 0 | 1 | 0.9947 | 0 | [A/G] | 372 | 2 |
| 96 | rs544345502 | 10 | 96561875 | 1 | 0.528 | 0 | 0 | 1 | 0.9973 | 0 | [T/C] | 373 | 1 |
| 97 | rs540418228 | 10 | 96561899 | 0 | 0.4122 | 0 | 0 | 0 | 0 | 0 | [A/G] | 0 | 374 |
| 98 | rs4494250 | 10 | 96563757 | 0.9871 | 0.8878 | 0.0455 | 0.3342 | 0.6203 | 1 | 0.2126 | [T/C] | 374 | 0 |
| 99 | rs141417293 | 10 | 96565286 | 1 | 0.8919 | 0 | 0.0053 | 0.9947 | 1 | 0.0027 | [A/C] | 374 | 0 |
| 100 | rs143612134 | 10 | 96565516 | 1 | 0.8841 | 0.9947 | 0.0053 | 0 | 1 | 0.0027 | [T/C] | 374 | 0 |
| 101 | rs150790215 | 10 | 96569418 | 0.9999 | 0.6878 | 0 | 0.016 | 0.984 | 1 | 0.008 | [T/C] | 374 | 0 |
| 102 | rs187091323 | 10 | 96573010 | 0.8991 | 0.3871 | 0.0027 | 0.0863 | 0.9111 | 0.992 | 0.0458 | [A/G] | 371 | 3 |
| 103 | rs2260946 | 10 | 96574659 | 0.2952 | 0.4806 | 0.7406 | 0.2246 | 0.0348 | 1 | 0.1471 | [A/G] | 374 | 0 |
| 104 | rs535259152 | 10 | 96574837 | 1 | 0.8117 | 0 | 0 | 1 | 1 | 0 | [A/C] | 374 | 0 |
| 105 | rs548874926 | 10 | 96574838 | 1 | 0.9062 | 1 | 0 | 0 | 1 | 0 | [T/A] | 374 | 0 |
| 106 | rs190777341 | 10 | 96575307 | 1 | 0.8698 | 1 | 0 | 0 | 1 | 0 | [C/G] | 374 | 0 |
| 107 | rs575923433 | 10 | 96575566 | 1 | 0.8929 | 0 | 0 | 1 | 1 | 0 | [T/C] | 374 | 0 |
| 108 | rs4417205 | 10 | 96580202 | 0.2277 | 0.9223 | 0.0348 | 0.2139 | 0.7513 | 1 | 0.1417 | [G/C] | 374 | 0 |
| 109 | rs559628884 | 10 | 96580264 | 1 | 0.859 | 0 | 0 | 1 | 1 | 0 | [T/G] | 374 | 0 |
| 110 | rs539248491 | 10 | 96580410 | 1 | 0.8207 | 1 | 0 | 0 | 1 | 0 | [T/C] | 374 | 0 |
| 111 | rs200003088 | 10 | 96580414 | 1 | 0.8484 | 0 | 0 | 1 | 1 | 0 | [T/C] | 374 | 0 |
| 112 | rs566054687 | 10 | 96586323 | 1 | 0.5089 | 1 | 0 | 0 | 0.9973 | 0 | [T/C] | 373 | 1 |
| 113 | rs555356783 | 10 | 96586732 | 1 | 0.5054 | 0 | 0 | 1 | 0.9973 | 0 | [A/G] | 373 | 1 |
| 114 | rs186575758 | 10 | 96587907 | 1 | 0.5937 | 0 | 0 | 1 | 1 | 0 | [T/G] | 374 | 0 |
| 115 | rs4917612 | 10 | 96591284 | 0.3478 | 0.7055 | 0.8342 | 0.1497 | 0.016 | 1 | 0.0909 | [G/C] | 374 | 0 |
| 116 | rs75105257 | 10 | 96593024 | 1 | 0.8788 | 1 | 0 | 0 | 1 | 0 | [A/G] | 374 | 0 |
| 117 | rs11188092 | 10 | 96597751 | 0.324 | 0.5378 | 0.6123 | 0.361 | 0.0267 | 1 | 0.2072 | [T/G] | 374 | 0 |
| 118 | rs11528090 | 10 | 96598439 | 0.9858 | 0.7644 | 0.6176 | 0.3369 | 0.0455 | 1 | 0.2139 | [T/G] | 374 | 0 |
| 119 | rs7915414 | 10 | 96599510 | 0.5091 | 0.7197 | 0.0598 | 0.3207 | 0.6196 | 0.984 | 0.2201 | [T/C] | 368 | 6 |
| 120 | rs28399513 | 10 | 96602398 | 0.2642 | 0.9192 | 0.0267 | 0.1898 | 0.7834 | 1 | 0.1217 | [T/A] | 374 | 0 |
| 121 | rs138142612 | 10 | 96602618 | 1 | 0.7909 | 0 | 0 | 1 | 1 | 0 | [A/G] | 374 | 0 |
| 122 | rs118203757 | 10 | 96602636 | 1 | 0.8795 | 0 | 0 | 1 | 0.9973 | 0 | [A/G] | 373 | 1 |
| 123 | rs770829708 | 10 | 96602653 | 1 | 0.8215 | 0 | 0.0027 | 0.9973 | 0.9973 | 0.0013 | [T/C] | 373 | 1 |
| 124 | rs201132803 | 10 | 96602666 | 1 | 0.8453 | 0 | 0 | 1 | 1 | 0 | [T/A] | 374 | 0 |
| 125 | rs144036596 | 10 | 96602710 | 1 | 0.8375 | 0 | 0 | 1 | 1 | 0 | [T/C] | 374 | 0 |
| 126 | rs550527959 | 10 | 96602711 | 1 | 0.9294 | 1 | 0 | 0 | 1 | 0 | [T/A] | 374 | 0 |
| 127 | rs771120274 | 10 | 96602769 | 1 | 0.5382 | 1 | 0 | 0 | 1 | 0 | [T/G] | 374 | 0 |
| 128 | rs11592737 | 10 | 96603414 | 0.3773 | 0.8743 | 0.6096 | 0.361 | 0.0294 | 1 | 0.2099 | [T/C] | 374 | 0 |
| 129 | rs4917623 | 10 | 96609568 | 0.9609 | 0.8877 | 0.3262 | 0.492 | 0.1818 | 1 | 0.4278 | [T/C] | 374 | 0 |
| 130 | rs368303159 | 10 | 96609654 | 0 | 0.4695 | 0 | 0 | 0 | 0 | 0 | [A/G] | 0 | 374 |
| 131 | rs185136199 | 10 | 96609663 | 1 | 0.8115 | 0 | 0 | 1 | 1 | 0 | [T/C] | 374 | 0 |
| 132 | rs55948420 | 10 | 96609704 | 1 | 0.8102 | 0 | 0 | 1 | 1 | 0 | [A/G] | 374 | 0 |
| 133 | rs144056033 | 10 | 96609740 | 1 | 0.8949 | 1 | 0 | 0 | 1 | 0 | [A/G] | 374 | 0 |
| 134 | rs17879685 | 10 | 96609752 | 1 | 0.7303 | 0 | 0.0027 | 0.9973 | 1 | 0.0013 | [T/C] | 374 | 0 |
| 135 | rs527499296 | 10 | 96609769 | 1 | 0.8053 | 1 | 0 | 0 | 1 | 0 | [T/G] | 374 | 0 |
| 136 | rs17886522 | 10 | 96609775 | 0 | 0.4685 | 0 | 0 | 0 | 0 | 0 | [A/C] | 0 | 374 |
| 137 | rs12268020 | 10 | 96612371 | 0.3773 | 0.8782 | 0.0294 | 0.361 | 0.6096 | 1 | 0.2099 | [A/G] | 374 | 0 |
| 138 | rs56337013 | 10 | 96612495 | 1 | 0.8796 | 0 | 0 | 1 | 1 | 0 | [T/C] | 374 | 0 |
| 139 | rs5787121 | 10 | 96612512 | 1 | 0.8404 | 0 | 0 | 1 | 1 | 0 | [D/I] | 374 | 0 |
| 140 | rs192154563 | 10 | 96612522 | 1 | 0.8179 | 0 | 0 | 1 | 1 | 0 | [A/G] | 374 | 0 |
| 141 | rs118203759 | 10 | 96612542 | 1 | 0.8212 | 1 | 0 | 0 | 1 | 0 | [C/G] | 374 | 0 |
| 142 | rs375283723 | 10 | 96612596 | 1 | 0.9013 | 0 | 0 | 1 | 1 | 0 | [A/G] | 374 | 0 |
| 143 | rs28399514 | 10 | 96612638 | 1 | 0.7822 | 0 | 0 | 1 | 1 | 0 | [A/G] | 374 | 0 |
| 144 | rs55640102 | 10 | 96612671 | 1 | 0.914 | 1 | 0 | 0 | 1 | 0 | [T/G] | 374 | 0 |
| 145 | rs733115 | 10 | 96619086 | 1 | 0.8877 | 0.866 | 0.128 | 0.053 | 1 | 0.069 | [G/T] | 374 | 0 |
| 146 | rs941890 | 10 | 96619606 | 1 | 8207 | 0.952 | 0.455 | 0.027 | 1 | 0.0254 | [G/A] | 374 | 0 |

**Table S2 The impact of detected *CYP2C19* and *CYP2C18* gene polymorphisms on human health and several drugs.**

| n | Variant | Gene | Drugs-related | Impact of SNP on the related drugs | Health disorder related | Clinical implications |
| --- | --- | --- | --- | --- | --- | --- |
| 1. | rs7900135 | *CYP2C18* | Torsemide | Impact on Torsemide PK/PD: Patients carrying minor allele of rs7900135 variant may slightly prolong the half-life of torsemide drug and could prolong the diuretic effect compared to wild-type allele carriers. | NA | NA |
| 2. | rs4986894 | *CYP2C19* | 4-tert-Octylphenol | Impact on 4-tert-Octylphenol PK/PD: Carriers of rs4986894 mutant allele C (CC or TC) had significant higher blood levels of 4-tert-Octylphenol with more cases of male infertility. | Male infertility | Male carriers of rs4986894 mutant allele C (CC or TC) had significant higher risk of being infertile when exposed to 4-tert-Octylphenol alkylphenol, which found in wastewater and fish. |
| 3. | rs4494250 | *CYP2C19* | Anti-HIV drugs (NNRTIs and PIs) | Impact on Anti-HIV drugs (NNRTIs and PIs) PK/PD: Patients carrying rs4494250 A allele may have better anti-HIV treatment efficacy by reducing viral load if they treated with NNRTIs and PIs drugs compared with those carrying GG genotype. | Breast cancer, high diastolic Blood pressure (DBP), dyslipidemia, and Chronic Obstructive Pulmonary Disease (COPD) | Increase Blood pressure (DBP):  rs4494250 A allele is linked to increase risk of raised diastolic blood pressure (DBP) compared to GG genotype.   Breast Cancer:  Rs4494250 minor allele may linked to breast cancer risk compared to wild-type allele.   Chronic Obstructive Pulmonary Disease (COPD):  rs4494250 minor allele may linked to COPD risk compared to wild-type allele.   Dyslipidemia (high triglyceride):  rs4494250 minor allele may linked to high TG risk compared to wild-type allele. |
| 4. | rs1326830 | *CYP2C18* | Atypical antipsychotics (quetiapine, aripiprazole, clozapine, olanzapine, and risperidone) | Impact on atypical antipsychotics PK/PD: Patients carrying minor allele of rs1326830 variant and using quetiapine, aripiprazole, clozapine, olanzapine, and risperidone were probably lost weight instead of gaining weight. Hence, these atypical antipsychotics cause weight gain as a side effect. | NA | NA |
| 5. | rs17885179 | *CYP2C19* | Celecoxib, fluconazole, omeprazole, fluoxetine, sertraline, and fluvoxamine | Impact on Celecoxib, fluconazole, omeprazole, fluoxetine, sertraline, and fluvoxamine PK/PD: An in-vitro study showed that allele C of the variant rs17885179 is associated with significant increased sensitivity (lower IC50) to celecoxib, omeprazole and fluvoxamine as compared to allele A (wild-type allele). Fluconazole also showed to be more sensitive for the C allele compared to A allele, yet, the difference in IC50 was not significant. | NA | NA |
| 6. | rs17878459 | *CYP2C19* | Celecoxib, fluoxetine, fluvoxamine, fluconazole, voriconazole, and clopidogrel | Based on in-vitro study:  Allele C of the variant rs17878459 is associated with increased sensitivity (lower IC50) to celecoxib, omeprazole, fluconazole or fluvoxamine as compared to allele G (wild-type allele).  Based on clinical study: Patients carrying of rs17878459 C allele have higher trough level (Cmin) of voriconazole and may have better antifungal response compared to those who carry G allele.   Impact on Clopidogrel PK/PD:  Based on current pharmacogenetic studies, patients carrying the rs17878459 C allele may not respond to clopidogrel due to poor platelet inhibition efficacy revealed among them. | NA | NA |
| 7. | rs7067866 | *CYP2C19* | Chlorcycloguanil | Impact on Chlorcycloguanil PK/PD: Patients with rs7067866 minor allele were associated with ultra metabolism of chlorocycloguanil antimalaria drug, which showed lower blood concentration compared to patients have wild-type allele. Yet, the yielded difference was insignificant. | NA | NA |
| 8. | rs12571421 | *CYP2C19* | Clopidogrel, citalopram, escitalopram, etravirine, tamoxifen | Impact on Clopidogrel PK/PD: Patients who carry minor allele of rs12571421 variant are at higher risk of clopidogrel resistance compared to wild-type allele carriers.   Impact on Citalopram and escitalopram PK/PD: Patients on either citalopram or escitalopram who carry minor allele of rs12571421 variant could have better antidepressant efficacy but higher risk of adverse events due to poor metabolism compared to wild-type allele carriers.   Impact on Etravirine PK/PD: HIV-infected patients on etravirine who carry minor allele of rs12571421 variant could have better anti-HIV efficacy but higher risk of adverse events due to poor metabolism compared to wild-type allele carriers.   Impact on Tamoxifen PK/PD: Women who carry minor allele of rs12571421 variant may have lower norendoxifen (aromatase inhibitor and active metabolite of tamoxifen) concentrations due to poor metabolism of tamoxifen compared to wild-type allele carriers. | NA | NA |
| 9. | rs7915414 | *CYP2C19* | Clopidogrel | Impact on Clopidogrel PK/PD:  rs7915414 variant A allele is significantly associated with a lack of clopidogrel efficacy as compared to the referenced allele (G allele). | NA | NA |
| 10. | rs12248560 | *CYP2C19* | Clopidogrel, prasugrel, impiramine, clomipramine, amitryptaline, citalopram, escitalopram, sertraline, voriconazole, lansoprazole, pantoprazole, omeprazole  stradiol, busulfan, cyclophosphamide, doxorubicin, fluorouracil, diazepam, phenazepam, 3,4-methylenedioxymethamphetamine, mephenytoin, progesterone, tamoxifen, leflunomide, warfarin, clozapine, chlorcycloguanil and estradiol | Impact on Clopidogrel and prasugrel PK/PD:  Carriers of the rs12248560 T allele had a significantly higher rate of hyper-response of clopidogrel and a higher rate of bleeding complications than patients carrying the CC genotype. For prasugrel, carriers of rs12248560 T allele had a significantly higher rate of hyper-response of prasugrel and a higher rate of bleeding complications than patients carrying the CC genotype.  Impact on Impiramine, clomipramine, and amitryptaline PK/PD:  Patients carrying the CYP2C19*17 allele in combination with a no or decreased function allele may have decreased metabolism of imipramine as compared to patients with two normal function alleles. This annotation only covers the pharmacokinetic relationship between CYP2C19 and imipramine and does not include evidence about clinical outcomes.   Impact on Citalopram, escitalopram, and sertraline PK/PD: Patients carrying the CYP2C19*17 allele in combination with a no or decreased function allele may have decreased metabolism of citalopram as compared to patients with two normal function alleles. However, conflicting evidence has been reported. This annotation only covers the pharmacokinetic relationship between CYP2C19 and citalopram and does not include evidence about clinical outcomes.  Impact on Voriconazole PK/PD: Patients carrying the CYP2C19*17 allele in combination with a no or decreased function allele may have decreased metabolism of voriconazole.  Impact on Lansoprazole, pantoprazole, and omeprazole PK/PD: Patients carrying the CYP2C19*17 allele in may have increased metabolism of lansoprazole as compared to patients with two normal function alleles.   Impact in Busulfan, Cyclophosphamide, doxorubicin, and fluorouracil PK/PD: Patients with the TT (CYP2C19 *17/*17) genotype undergoing transplantation may have increased metabolism of busulfan as compared to patients with the CC (*1/*1) genotype. However, some contradictory evidence exists for this association. Cyclophosphamide, doxorubicin, fluorouracil: Patients with the TT genotype may have a decreased risk for leukopenia adverse event when treated with cyclophosphamide, doxorubicin and fluorouracil (FAC) as compared to patients with CC genotype.   Impact on Diazepam and Phenazepam PK/PD: Patients with the TT genotype (*17/*17) may have a decreased response and fewer adverse events to diazepam and phenazepam as compared to patients with the CC (*1/*1) or CT (*1/*17) genotypes.  Impact on 3,4-methylenedioxymethamphetamine PK/PD: People with an ultra-rapid metabolizer genotype (e.g. *17/*17) may have increased metabolism and lesser risk of hypertension of 3,4-methylenedioxymethamphetamine compared to people with intermediate metabolizer genotypes.  Impact on Mephenytoin PK/PD: Patients with the TT genotype who are treated with mephenytoin may require an increased dose as compared to patients with the CC genotype.  Impact on Progesterone PK/PD: Women with the T allele who are treated with progesterone oral and vaginal dosage forms have lower values of progesterone AUC and Cmax, thus, may require an increased dose or dose frequency as compared to women with the CC genotype.  Impact on Tamoxifen PK/PD:  Women with breast cancer and the CYP2C19 *1/*17 or *17/*17 genotype who are treated with tamoxifen may have poorer response to tamoxifen as compared to those with the *1/*1 genotype.   Impact on Leflunomide PK/PD: Patients carrying CYP2C19 *1/*17 or *17/*17 genotype who are treated with leflonomide may experience less side effects and less leflunomide cessation compared to those with the *1/*1, *1/*2, and *2/*2 genotypes.   Impact on Warfarin PK/PD: Warfarin may be less effective or require higher doses among patients carrying CYP2C19 *1/*17 or *17/*17 genotype compared to those with the *1/*1, *1/*2, and *2/*2 genotypes.   Impact on Clozapine PK/PD:  Clozapine is less effective among patients with schizophrenia carrying CYP2C19 *1/*17 or *17/*17 genotype and may lead to refractory schizophrenia but less adverse events compared to those with the *1/*1, *1/*2, and *2/*2 genotypes.   Impact on Estradiol PK/PD: No studies clearly mentioned drugs that associated with rs12248560 variant. Only estradiol could be related due to endometriosis and breast cancer association. | Endometriosis, breast cancer, Tetralogy of Fallot, Major depressive disorder, Hypertension, and Type-2 Diabetes Mellitus | Endometriosis:  Women carrying rs12248560 T allele (CT and TT genotypes) are at lower risk of endometriosis, which could be related to the increased catabolism of estradiol.   Breast cancer:  Women carrying rs12248560 T allele (CT and TT genotypes) are at lower risk of breast cancer, which could be related to the increased catabolism of estradiol.  Tetralogy of Fallot (birth defect that affects normal blood flow through the heart): Pregnant women carrying rs12248560 T allele (CT and TT genotypes) and might be at higher risk of delivering newborns with tetralogy of Fallot (birth defect that affects normal blood flow through the heart) when the pregnant women exposed to high Nitrous dioxide (NO2) pollutant during first eight weeks of pregnancy.   Major depressive disorder: Patients carrying rs12248560 CC genotype showed a six‐fold higher likelihood of clinically regular antidepressant therapy at follow‐up with no new depression episode detected (more stable cases) compared to minor allele homozygotes [TT; ultrarapid metabolizers].  Hypertension: People carrying rs12248560 T allele (CT and TT genotypes) are at higher risk of having hypertension compared with those who have rs12248560 CC genotype.  Type-2 Diabetes Mellitus: People carrying rs12248560 TT genotype are at higher risk of having type-2 diabetes compared with those who have rs12248560 C allele (CC and CT genotypes). |
| 10. | rs12248560 | *CYP2C19* |  |  |  |  |
| 10. | rs12248560 | *CYP2C19* |  |  |  |  |
| 11. | rs1126545 | *CYP2C18* | Clozapine | Impact on Clozapine PK/PD: Allele T is associated with decreased metabolism and more adverse events of clozapine in people with Schizophrenia as compared to allele C. | NA | NA |
| 12. | rs7916649 | *CYP2C19* | Clozapine, valproic acid, and clopidogrel | Impact on Valproic acid PK/PD: The A allele at rs7916649 (G>A) in CYPC19 was associated with 1.95 (1.37-2.76) times increased risk for daily dose reduction of valproic acid.   Impact on Clopidogrel PK/PD: People having rs7916649 allele are at significant higher risk of recurrent cardiac event due to treatment failure.   Impact on Clozapine PK/PD: People having rs7916649 minor allele may have more side effects of clozapine compared to those carrying wile type allele. | NA | NA |
| 13. | rs11188059 | *CYP2C18* | Escitalopram, sertraline, voriconazole, and omeprazole | Impact on Escitalopram and sertraline PK/PD: Allele G is associated with decreased concentrations of escitalopram and sertraline as compared to allele A.  Impact on Voriconazole PK/PD: Rs11188059 allele G is associated with decreased trough concentrations of voriconazole in people with Leukemia, Lymphoma or Myelodysplastic Syndromes as compared to rs11188059 A allele.  Impact on Omeprazole PK/PD:  rs11188059 and rs2860840) are associated with increased enzymatic activity, and may reduce omeprazole exposure. | NA | NA |
| 14. | rs12769205 | *CYP2C19* | Imipramine, clomipramine, trimipramine, amitriptyline and doxepin, lansoprazole, dexlansoprazole, pantoprazole, rabeprazole, esomeprazole, omeprazole, clopidogrel, voriconazole, citalopram, escitalopram, sertraline, tamoxifen, tacrolimus, 3,4-Methylenedioxymethamphetamine (MDMA), glyburide, gliclazide, glimepiride, glipizide, icotinib, metamizole, brivaracetam, clobazam, diazepam, methylphenobaribtal, mephenytoin, warfarin, bupropion, venlavaxine, and progesterone, methadone, valproic acid, and carisoprodol | Impact on Imipramine, clomipramine, trimipramine, amitriptyline and doxepin PK/PD: Pharmacokinetic studies showed that tricyclic antidepressants, including imipramine, clomipramine, trimipramine, amitriptyline and doxepin were poorly metabolized and their blood level were increased among carriers of rs12769205 mutant allele G (GG or AG). In addition, the CPIC Dosing Guideline update recommends to consider a 50% dose reduction in carriers of rs12769205 mutant allele G.   Impact on Lansoprazole, dexlansoprazole, pantoprazole, rabeprazole, esomeprazole, and omeprazole PK/PD: Patients carrying the rs12769205 mutant allele G may have a better response to proton pump inhibitors (PPI), including lansoprazole, dexlansoprazole, pantoprazole, rabeprazole, esomeprazole, and omeprazole as compared to patients with two normal function alleles.  Impact on Clopidogrel PK/PK: There is an increase in platelet reactivity among patients carrying the rs12769205 mutant allele G. Based on current pharmacogenetic studies, patients carrying the rs12769205 mutant allele G may not respond to clopidogrel due to poor platelet inhibition efficacy revealed among them. However, genotyping for CYP2C19 rs12769205 mutant allele is not recommended when considering clopidogrel treatment for patients, as some trials showed specific non-genetic factors (e.g. age and diabetes) that could affect clopidogrel responsiveness. Instead, platelets inhibition tests could be used as predictors of the clinical efficacy of clopidogrel treatment. Other P2Y12 receptor inhibitors should be considered as alternative medications.   Impact on Voriconazole PK/PD: Patients carrying the rs12769205 mutant allele G have higher risk of drug-related hepatotoxicity and neurotoxicity events.  Impact on Citalopram, escitalopram, and sertraline PK/PD: Users of citalopram, escitalopram, or sertraline carrying rs12769205 mutant allele G are at more risk of having adverse drug reactions, such as gastrointestinal symptoms and sexual dysfunction.   Impact on Tamoxifen PK/PD:  The duration of clinical responsiveness of tamoxifen was significantly common among breast cancer patients carrying rs12769205 mutant allele G, which indicates higher efficacy of tamoxifen in this group of patients.    Impact on Tacrolimus PK/PD:  Patients with kidney transplant carrying rs12769205 mutant GG genotype are associated with significant higher concentrations of tacrolimus compared with AA and AG genotypes.   Impact on MDMA PK/PD:  People carrying rs12769205 mutant G allele and taking MDMA experienced higher heart rate and higher systolic blood pressure compared with individuals carrying wild-type.   Impact on Glyburide, gliclazide, glimepiride, and glipizide PK/PD:  Patients carrying rs12769205 mutant G allele may have increased response to sulfonylureas, including glyburide, gliclazide, glimepiride, and glipizide (reduced risk of sulfonylureas treatment failure and better HbA1c response) as compared to patients carrying two CYP2C19 functional alleles.   Impact on Icotinib PK/PD:  A study showed that the pharmacokinetics of icotinib differ significantly between carriers of rs12769205 GG genotype and those carriers of AG and AA genotypes. Individuals with rs12769205 GG genotype had higher icotinib blood concentration and one patient experienced urticaria.  Impact on Metamizole PK/PD:  Patients with rs12769205 G allele may have decreased metabolism of metamizole as compared to patients carrying the AA genotype. Therefore, toxicity risk of metamizole (e.g., agranulocytosis) could be increased among CYP2C19 poor metabolizers.    Patients with rs12769205 G allele have significant decrease in the metabolism of brivaracetam, clobazam, diazepam, methylphenobaribtal, and mephenytoin drugs.  Impact on Warfarin PK/PD:  Patients with the rs12769205 G allele may require a decreased dose of warfarin as compared to patients with the AA genotype. Hence, rs12769205 G allele may associated with increased risk of bleeding.   Impact on Bupropion, venlavaxine PK/PD:  Patients with rs12769205 G allele have significant decrease in the metabolism of bupropion and venlafaxine drugs, which may induce more adverse drug reactions.   Impact on Progesterone PK/PD:  Women carrying rs12769205 G allele showed significant higher adjusted AUCt and adjusted Cmax than AA genotype.   Impact on Methadone PK/PD:  Patients carrying the rs12769205 G allele in combination with a normal function allele may have increased concentrations of methadone as compared to patients with two normal functional alleles. However, multiple studies have failed to find this association.   Impact on Valproic acid PK/PD:  Women carrying rs12769205 G allele and epilepsy who are taking valproic acid may have increased risk of becoming overweight compared to patients with normal metabolizer genotypes. However, this result was not found in men or in another study.   Impact on Carisoprodol PK/PD:  Healthy individuals carrying rs12769205 G allele may have increased concentrations of carisoprodol as compared to those with functional CYP2C19 alleles. | NA | NA |
| 14. | rs12769205 | *CYP2C19* |  |  |  |  |
| 14. | rs12769205 | *CYP2C19* |  |  |  |  |
| 14. | rs12769205 | *CYP2C19* |  |  |  |  |
| 14. | rs12769205 | *CYP2C19* |  |  |  |  |
| 15. | rs145119820 | *CYP2C19* | Mephenytoin | Impact on Mephenytoin PK/PD: Carriers of rs145119820 variant with A allele are associated with decreased clearance of mephenytoin as compared to those carrying allele G. Besides, Devarajan et al. (2019) showed that mephynytoin was more sensitive (lower Km (Michaelis constant)) and less reacted with CYP2C19 enzyme in A allele carriers compared to G allele. | NA | NA |
| 16. | rs11528090 | *CYP2C19* | Nevirapine | Impact on Nevirapine PK/PD: rs11528090 G allele may associated with higher trough plasma concentration of nevirapine and could lead to better efficacy but more side effects. | NA | NA |
| 17. | rs4388808 | *CYP2C19* | Nevirapine | Impact on Nevirapine PK/PD:  rs4388808 G allele may associated with higher trough plasma concentration of nevirapine and could lead to better efficacy but more side effects. | Alzheimer disease | Alzheimer disease: People carrying rs4388808 G allele are significantly (p=0.0006) associated with a lower amyloid-β Aβ load in the frontal, inferior temporal, and posterior cingulate cortices in the brain compared with AA genotype. Therefore, rs4388808 G allele recognized as a protective genetic factor against Alzheimer disease. |
| 18. | rs12768009 | *CYP2C19* | Nevirapine and pesticides | Impact on Nevirapine PK/PD: rs12768009 genotype AA is associated with decreased clearance of nevirapine in people with HIV Infections as compared to genotypes AG + GG.  Impact on Pesticides PK/PD: Men carrying rs12768009 minor alleles are at higher risk of prostate cancer when exposed to pesticides compared to carriers of wild-type allele. This risk is due to poor metabolizing of pesticides and exposed to their toxicities for longer period. | Prostate cancer | Prostate cancer:  Men carrying rs12768009 minor alleles are at higher risk of prostate cancer when exposed to pesticides compared to carriers of wild-type allele. This risk is due to poor metabolizing of pesticides and exposed to their toxicities for longer period. |
| 19. | rs11188072 | *CYP2C19* | Omeprazole, clopidogrel, mephenytoin, imipramine, citalopram, and escitalopram | Impact on Omeprazole PK/PD:  Patients carrying rs11188072 T allele associated with higher risk of omeprazole treatment failure or required higher doses of omeprazole.  Impact on Clopidogrel PK/PD:  Patients carrying rs11188072 T allele might be at lower risk of cardiovascular event due to increase clopidogrel response and better antiplatelet action. However, a meta-analysis conducted by Bauer et al. revealed insignificant difference among carriers of different rs11188072 genotypes.   Impact on Mephenytoin PK/PD:  Mephenytoin blood concentration was extremely lower among people carrying rs11188072 TT genotype compared to those with wile-type genotype.   Impact on Imipramine PK/PD:  Patients carrying rs11188072 T allele may associated with higher risk of imipramine treatment failure, untreated depressive episodes, or required higher doses of imipramine.  Impact on Citalopram and Escitalopram PK/PD:  Patients carrying rs11188072 T allele may associated with higher risk of citalopram and escitalopram treatment failure, low chance of depression remission, or required higher doses. | Endometrium thickness among postmenuposal women taking tamoxifen | Endometrial thickness among postmenopausal women using tamoxifen: Postmenopausal women using tamoxifen and carrying rs11188072 TT genotype associated with higher risk of having more thicker endometrium. |
| 20. | rs2860840 | *CYP2C18* | Omeprazole, escitalopram, sertraline, voriconazole. and warfarin | Impact on Omeprazole PK/PD: Patients with rs2860840 allele T are associated with decreased concentrations of omeprazole, which linked to treatment failure and may require higher doses as compared to patients carrying CC genotype.  Impact on Escitalopram and sertraline PK/PD: rs2860840 allele T is associated with decreased concentrations of escitalopram and sertraline as compared to allele C.  Impact on Voriconazole PK/PD: Patients with rs2860840 allele T are associated with decreased voriconazole trough concentrations, which linked to treatment failure and may require higher doses as compared to patients carrying CC genotype.  Impact on Warfarin PK/PD: Patients carrying rs2860840 T allele may linked to decreased warfarin efficacy due to rapid metabolism compared to patients carrying CC genotype. | NA | NA |
| 20. | rs2860840 | *CYP2C18* |  |  |  |  |
| 21. | rs770829708 | *CYP2C19* | Omeprazole, mephenytoin, voriconazole, fluoxetine, and methadone | Based on in-vitro studies: rs770829708 with A allele is associated with decreased clearance of voriconazole, methadone, omeprazole, mephenytoin, and fluoxetine as compared to allele G. | NA | NA |
| 22. | rs17884832 | *CYP2C19* | Pesticide and clopidogrel | Impact on pesticide PK/PD:  People carrying minor allele (G allele) of the rs17884832 variant are at significantly lower risk of insufficient detoxification of pesticide exposed compared to T allele carriers. Therefore, Carriers of rs17884832 variant minor allele at lower risk of pesticide toxicity, such as cardiovascular disease, allergies, and gastrointestinal disorders.  Impact on clopidogrel PK/PD:  People having rs17884832 minor allele and using clopidogrel are at significant higher risk of recurrent cardiac event due to treatment failure compared with those have wild type allele. | NA | NA |
| 23. | rs11188092 | *CYP2C19* | Pesticides | Impact on pesticide PK/PD:  People carrying minor alleles (C or T alleles) of the rs11188092 variant are at higher risk of insufficient detoxification of pesticide exposed compared to A allele carriers. Insufficient detoxification of pesticide may lead to cardiovascular disease, allergies, and gastrointestinal disorders. | NA | NA |
| 24. | rs4917623 | *CYP2C19* | Tamoxifen, tacrolimus, nevirapine, and praziquantel | Impact on Tamoxifen PK/PD: Patients carrying rs4917623 TC and CC genotypes of the CYP2C19 gene have enhanced anti-cancer efficacy for longer periods compared to the TT genotype. However, the difference was insignificant.   Impact on Tacrolimus PK/PD: The pharmacokinetics of tacrolimus in patients carrying rs4917623 variant showed a significant variation compared to CYP2C19 wild-type allele carriers.  Impact on Nevirapine PK/PD: Carriers of rs4917623 C allele were associated with higher trough levels of nevirapine. In addition, those patients carrying the rs4917623 C allele showed better efficacy and higher frequency of ADRs compared to patients carrying the rs4917623 TT genotype.  Impact on Praziquantel PK/PD:  Patients carrying the TC rs4917623 genotype compared to the TT genotype. Yet, this higher efficacy was insignificant. | Breast cancer | Breast cancer: CYP2C19 rs4917623 heterozygous genotype was highly associated with breast cancer (OR 1.38) than wild-type homozygous genotype carriers. |
| 25. | rs7917985 | *CYP2C18* | Tenofovir | Impact on Tenofovir PK/PD: Patients carrying minor allele of rs7917985 variant have elevated blood concentration of tenofovir compared to those carrying wikd-type, however, the difference was insignificant. | NA | NA |
| 26. | rs2901783 | *CYP2C18* | Clopidogrel and warfarin | Impact on Clopidogrel PK/PD:  rs2901783 variant is significantly associated with a lack of clopidogrel efficacy as compared to the referenced allele.  Impact on Warfarin PK/PD: rs2901783 was associated with warfarin sensitivity; patients who were homozygous wild-type (AA) for this SNP achieved therapeutic INR the fastest, the homozygous variant genotype (GG or TT) group took the longest, with heterozygotes occupying an intermediate position. | NA | NA |
| 27. | rs7085563 | *CYP2C18* | Warfarin | Impact on Warfarin PK/PD:  People carrying rs11188092 A allele are at higher risk of warfarin plasma concentration variations compared to wild-type allele. | NA | NA |
| 28. | rs3814637 | *CYP2C19* | Warfarin and anlotinib | Impact on warfarin PK/PD: rs3814637 TT genotype may associated with increased risk of over-anticoagulation as compared to with the CC genotype.  Impact on anlotinib PK/PD: The peak plasma concentrations of anlotinib among rs3814637 mutant allele T carriers (TT or CT) were significantly higher than those of wildtypes (CC). | NA | NA |
| 29. | rs4417205 | *CYP2C19* | Clopidogrel | Impact on Clopidogrel PK/PD: Patients on clopidogrel who carry rs4417205 variant are significantly showed less anti-platelet efficacy compared with those who carry wild-type allele. | NA | NA |
| 30. | rs11592737 | *CYP2C19* | NA | NA | Endometriosis and infertility among women with endometriosis | Endometriosis:  Women carrying the rs11592737 GG genotype are at higher risk of having endometriosis and being infertile compared with those who have the rs11592737 A allele.   Infertility among women with endometriosis: Women carrying rs11592737 GG genotype are at higher risk of having Infertile women with endometriosis compared with those who have rs11592737 A allele. |
| 31. | rs17879685 | *CYP2C19* | NA | NA | NA | NA |
| 32. | rs12268020 | *CYP2C19* | NA | NA | NA | NA |
| 33. | rs2260946 | *CYP2C19* | NA | NA | NA | NA |
| 34. | rs932809 | *CYP2C18* | NA | NA | NA | NA |
| 35. | rs28399513 | *CYP2C19* | NA | NA | NA | NA |
| 36. | rs3740367 | *CYP2C18* | NA | NA | NA | NA |
| 37. | rs117140172 | *CYP2C18* | NA | NA | NA | NA |
| 38. | rs7902257 | *CYP2C19* | NA | NA | NA | NA |
| 39. | rs17879992 | *CYP2C19* | NA | NA | NA | NA |
| 40. | rs187091323 | *CYP2C19* | NA | NA | NA | NA |
| 41. | rs4917612 | *CYP2C19* | NA | NA | NA | NA |
| 42. | rs733115 | *CYP2C19* | NA | NA | NA | NA |
| 43. | rs941890 | *CYP2C19* | NA | NA | NA | NA |
| 44. | rs150579865 | *CYP2C18* | NA | NA | NA | NA |
| 45. | rs60181876 | *CYP2C18* | NA | NA | NA | NA |
| 46. | rs59636573 | *CYP2C18* | NA | NA | NA | NA |
| 47. | rs113164681 | *CYP2C19* | NA | NA | NA | NA |
| 48. | rs111490789 | *CYP2C19* | NA | NA | NA | NA |
| 49. | rs17878739 | *CYP2C19* | NA | NA | NA | NA |
| 50. | rs17878649 | *CYP2C19* | NA | NA | NA | NA |
| 51. | rs141417293 | *CYP2C19* | NA | NA | NA | NA |
| 52. | rs143612134 | *CYP2C19* | NA | NA | NA | NA |
| 53. | rs150790215 | *CYP2C19* | NA | NA | NA | NA |

**PK/PD: Pharmacokinetics or pharmacodynamics. NA: No relevant data or information is available. NNRTIs and PIs: Non-nucleoside reverse transcriptase inhibitors and protease inhibitors.**

**Table S3 MAF of the measured variants (N=141) among Saudi participants compared with global and other ethnic groups’ outcomes*.**

| n | | Variant name | | Variant type | Nitrogenous bases change | | Gene | | Star Allele | MAF in the current study, % | | | Global MAF, % | MAF in Europeans, % | MAF in Africans, % | MAF in East Asians, % | MAF in South Asians, % | | MAF in Latin American, % | |
| --- | --- | --- | --- | --- | --- | --- | --- | --- | --- | --- | --- | --- | --- | --- | --- | --- | --- | --- | --- | --- |
| 1. | rs7067866 | | Intronic | | G>T | CYP2C19 | | - | | | 46.79 | 46.538 | | 44.329 | 64.42 | 43.33 | | 62.31 | | 28.9 |
| 2. | rs7916649 | | Intronic | | G>A | CYP2C19 | | CYP2C19*2B | | | 46.66 | 44.175 | | 43.5107 | 64.26 | 44.14 | | 60.85 | | 26.78 |
| 3. | rs4917623 | | Intronic | | T>C | CYP2C19 | | - | | | 42.78 | 50.617 | | 51.7452 | 23.12 | 54.53 | | 36.19 | | 65.85 |
| 4. | rs7917985 | | Intronic | | C>T | CYP2C18 | | - | | | 23.53 | 19.139 | | 20.31 | 22.74 | 6.92 | | 16.31 | | 9.89 |
| 5. | rs2901783 | | Intronic | | A>G | CYP2C18 | | - | | | 22.06 | 22.548 | | 21.7969 | 31.71 | 43.14 | | 47.42 | | 15.71 |
| 6. | rs7915414 | | Intronic | | G>A | CYP2C19 | | - | | | 22.01 | 23.279 | | 21.228 | 32.02 | 41.79 | | 47.17 | | 15.5 |
| 7. | rs11528090 | | Intronic | | T>G | CYP2C19 | | - | | | 21.43 | 18.436 | | 18.516 | 14.7 | 38.63 | | 12.65 | | 17.1 |
| 8. | rs4494250 | | Intronic | | G>A | CYP2C19 | | - | | | 21.26 | 33.402 | | 34.5741 | 6.65 | 18.76 | | 23.54 | | 50.74 |
| 9. | rs2860840 | | Intronic | | C>T | CYP2C18 | | - | | | 21.12 | 33.063 | | 34.8005 | 7.74 | 18.79 | | 23.46 | | 51.99 |
| 10. | rs12248560 | | Intronic | | C>T | CYP2C19 | | CYP2C19*17 | | | 20.99 | 22.229 | | 23.135 | 22.64 | 1.54 | | 14.81 | | 12 |
| 11. | rs11188072 | | Intronic | | C>T | CYP2C19 | | CYP2C19*17 | | | 20.99 | 20.012 | | 22.759 | 23.42 | 1.54 | | 14.73 | | 9.57 |
| 12. | rs12268020 | | Intronic | | C>T | CYP2C19 | | - | | | 20.99 | 20.672 | | 21.3206 | 22.41 | 1.54 | | 14.81 | | 10.19 |
| 13. | rs11592737 | | Intronic | | A>G | CYP2C19 | | - | | | 20.99 | 20.938 | | 21.6155 | 23.29 | 1.54 | | 14.81 | | 10.43 |
| 14. | rs4388808 | | Intronic | | A>G | CYP2C19 | | - | | | 20.99 | 17.314 | | 17.2113 | 13.78 | 35.15 | | 12.65 | | 18.71 |
| 15. | rs11188092 | | Intronic | | A>C | CYP2C19 | | - | | | 20.72 | 19.053 | | 21.587 | 22.69 | 1.54 | | 14.81 | | 9.39 |
| 16. | rs7085563 | | Intronic | | T>A | CYP2C18 | | - | | | 16.58 | 17.395 | | 14.91 | 28.31 | 37.69 | | 37.02 | | 11.4 |
| 17. | rs2260946 | | Intronic | | T>C | CYP2C19 | | - | | | 14.71 | 4.771 | | 3.998 | 7.92 | 20.83 | | 15.4 | | 2.2 |
| 18. | rs4417205 | | Intronic | | C>G | CYP2C19 | | CYP2C19*35 | | | 14.17 | 22.390 | | 14.22 | 19.04 | 32.22 | | 35.52 | | 11.2 |
| 19. | rs12769205 | | Intronic | | A>G | CYP2C19 | | CYP2C19*2E | | | 14.17 | 14.643 | | 14.225 | 19.82 | 32.22 | | 35.52 | | 10.37 |
| 20. | rs7900135 | | Intronic | | A>G | CYP2C18 | | - | | | 14.04 | 15.285 | | 14.528 | 20.58 | 32.22 | | 35.52 | | 11.1 |
| 21. | rs932809 | | Intronic | | C>T | CYP2C18 | | - | | | 14.04 | 15.168 | | 14.8395 | 20.01 | 32.22 | | 35.52 | | 11.12 |
| 22. | rs28399513 | | Intronic | | T>A | CYP2C19 | | - | | | 12.17 | 15.392 | | 14.536 | 18.6 | 28.63 | | 35.52 | | 10.8 |
| 23. | rs12768009 | | Intronic | | G>A | CYP2C19 | | - | | | 12.17 | 14.302 | | 14.1963 | 14.29 | 37.61 | | 36.94 | | 9.71 |
| 24. | rs12571421 | | Intronic | | A>G | CYP2C19 | | CYP2C19*2 | | | 11.36 | 15.522 | | 14.9232 | 17.31 | 32.22 | | 30.85 | | 12.46 |
| 25. | rs1126545 | | Exonic - Missense (T385M) | | C>T | CYP2C18 | |  | | | 11.36 | 14.825 | | 14.2795 | 14.3 | 30.29 | | 35.52 | | 8.97 |
| 26. | rs4986894 | | Intronic | | T>C | CYP2C19 | | CYP2C19*2D | | | 11.36 | 14.085 | | 14.1926 | 13.36 | 67.78 | | 64.48 | | 10.01 |
| 27. | rs4917612 | | Intronic | | C>G | CYP2C19 | | - | | | 9.09 | 6.241 | | 7.647 | 0.57 | 38.55 | | 9.57 | | 11 |
| 28. | rs3814637 | | Intronic | | C>T | CYP2C19 | | CYP2C19*15 | | | 7.91 | 3.455 | | 3.831 | 0.59 | 9.57 | | 11.65 | | 3.83 |
| 29. | rs17879992 | | Intronic | | T>A | CYP2C19 | | - | | | 7.37 | 7.088 | | 6.475 | 9.47 | 9.49 | | 11.56 | | 4.3 |
| 30. | rs17884832 | | Intronic | | T>G | CYP2C19 | | - | | | 6.95 | 6.461 | | 6.474 | 8.96 | 9.49 | | 11.56 | | 4.18 |
| 31. | rs733115 | | Unknown | | G>T | CYP2C19 | | - | | | 6.95 | 6.582 | | 6.2612 | 7.362 | 9.49 | | 11.56 | | 4.02 |
| 32. | rs3740367 | | Intronic | | G>A | CYP2C18 | | - | | | 4.81 | 6.512 | | 6.684 | 3.176 | 4.1 | | 10.15 | | 4 |
| 33. | rs187091323 | | Intronic | | A>G | CYP2C19 | | - | | | 4.58 | 0.104 | | 0.133 | 0 | 0.17 | | 0 | | 0 |
| 34. | rs11188059 | | Intronic | | G>A | CYP2C18 | | - | | | 4.55 | 13.332 | | 13.8327 | 2.73 | 0.26 | | 8.24 | | 16.65 |
| 35. | rs941890 | | Unknown | | G>A | CYP2C19 | | - | | | 2.54 | 1.943 | | 0.038 | 12.67 | 0 | | 0 | | 1.1 |
| 36. | rs7902257 | | Intronic | | G>A | CYP2C19 | | CYP2C19*27 | | | 2.28 | 0.410 | | 0.0269 | 7.24 | 0.17 | | 0 | | 0.39 |
| 37. | rs117140172 | | Intronic | | G>A | CYP2C18 | | - | | | 2.01 | 1.725 | | 1.907 | 0.06 | 0 | | 0.33 | | 1.9 |
| 38. | rs1326830 | | Intronic | | C>A | CYP2C18 | | - | | | 2.01 | 1.464 | | 0.7715 | 0.24 | 22.05 | | 18.47 | | 3.19 |
| 39. | rs113164681 | | Intronic | | C>T | CYP2C19 | | - | | | 0.94 | 0.091 | | 0 | 0.45 | 0 | | 0 | | 0 |
| 40. | rs17878739 | | Intronic | | T>C | CYP2C19 | | CYP2C19*15 | | | 0.94 | 0.409 | | 0.03 | 1.9 | 0 | | 0 | | 0 |
| 41. | rs145119820 | | Exonic - Missense (V113I) | | G>A | CYP2C19 | | CYP2C19*2 | | | 0.80 | 0.016 | | 0.0055 | 0 | 0 | | 0 | | 0.1 |
| 42. | rs150790215 | | Intronic | | G>A | CYP2C19 | | - | | | 0.80 | 0.028 | | 0 | 0.14 | 0 | | 0 | | 0 |
| 43. | rs17878459 | | Exonic - Missense (E92D) | | G>A | CYP2C19 | | CYP2C19*2B | | | 0.67 | 2.669 | | 2.901 | 1.18 | 0 | | 0 | | 2.2 |
| 44. | rs60181876 | | Exonic - Missense (T299I) | | C>T | CYP2C18 | | - | | | 0.67 | 0.067 | | 0.002 | 1.38 | 0 | | 0 | | 0.2 |
| 45. | rs150579865 | | Intronic | | G>T | CYP2C18 | | - | | | 0.53 | 0.049 | | 0.01 | 0.07 | 0 | | 0 | | 0.2 |
| 46. | rs111490789 | | Intronic | | C>A | CYP2C19 | | CYP2C19*28 | | | 0.41 | 0.409 | | 0.03 | 1.9 | 0 | | 0 | | 0 |
| 47. | rs17885179 | | Exonic -Missense (E122A) | | A>C | CYP2C19 | | CYP2C19*39 | | | 0.40 | 0.062 | | 0.005 | 0.84 | 0 | | 0 | | 0.095 |
| 48. | rs17878649 | | Intronic | | G>A | CYP2C19 | | - | | | 0.27 | 0.126 | | 0.0111 | 1.12 | 5.38 | | 1.41 | | 0.2 |
| 49. | rs141417293 | | Intronic | | C>A | CYP2C19 | | - | | | 0.27 | 0.007 | | 0 | 0.03 | 0 | | 0 | | 0 |
| 50. | rs143612134 | | Intronic | | T>C | CYP2C19 | | - | | | 0.27 | 0.361 | | 0.01 | 1.56 | 0 | | 0 | | 0 |
| 51. | rs770829708 | | Exonic - Missense (D341H) | | G>A | CYP2C19 | | CYP2C19*2F | | | 0.13 | 0.011 | | 0.015 | 0 | 0.02 | | 0.02 | | 0 |
| 52. | rs17879685 | | Exonic - Missense (R410C) | | C>T | CYP2C19 | | CYP2C19*13 | | | 0.13 | 0.092 | | 0.0016 | 1.603 | 0 | | 0 | | 0 |
| 53. | rs59636573 | | Exonic - Missense (V330L) | | G>T | CYP2C18 | | - | | | 0.13 | 0.057 | | 0.001 | 1.157 | 0 | | 0 | | 0.1 |
| 54. | rs116992754 | | Intronic | | G>T | CYP2C19 | | - | | | 0 | 1.11 | | 2.53 | 0.45 | 0 | | 0.67 | | 2.3 |
| 55. | rs117111102 | | Exonic - Missense (R124G) | | C>T | CYP2C18 | | - | | | 0 | 1.2852 | | 1.3368 | 0.34 | 1.58 | | 0.33 | | 0.5 |
| 56. | rs150562893 | | Intronic | | A>G | CYP2C18 | | - | | | 0 | 0.731 | | 0.854 | 0.17 | 0 | | 0 | | 0.7 |
| 57. | rs183718557 | | Intronic | | G>A | CYP2C18 | | - | | | 0 | 0.19 | | 0 | 0.08 | 0.79 | | 0.17 | | 0 |
| 58. | rs201633987 | | Intronic | | G>A | CYP2C18 | | - | | | 0 | 0.17 | | 0 | 0 | 0.85 | | 0.08 | | 0 |
| 59. | rs11188056 | | Exonic - Silent (L170L) | | C>T | CYP2C18 | | - | | | 0 | 0 | | 0 | 0 | 0 | | 0 | | 0 |
| 60. | rs141271146 | | Exonic - Nonsense (R186Ter) | | C>T | CYP2C18 | | - | | | 0 | 0.0043 | | 0.003 | 0.010 | 0 | | 0 | | 0 |
| 61. | rs115269792 | | Exonic - Missense (Q192H) | | G>C | CYP2C18 | | - | | | 0 | 0.16 | | 0 | 0 | 0.85 | | 0 | | 0 |
| 62. | rs148902102 | | Intronic | | T>G | CYP2C18 | | - | | | 0 | 1.26 | | 0 | 0 | 6.75 | | 0.17 | | 0 |
| 63. | rs2296681 | | Exonic - Missense (K232E) | | A>G | CYP2C18 | | - | | | 0 | 0.0036 | | 0 | 0 | 0.25 | | 0 | | 0 |
| 64. | rs184571563 | | Exonic - Missense (R261W) | | C>T | CYP2C18 | | - | | | 0 | 0.02 | | 0 | 0 | 0 | | 0 | | 0.1 |
| 65. | rs560806209 | | Intronic | | C>T | CYP2C18 | | - | | | 0 | 0.33 | | 0 | 0 | 0 | | 1.75 | | 0 |
| 66. | rs186024974 | | Intronic | | A>G | CYP2C18 | | - | | | 0 | 0.33 | | 0 | 0 | 1.71 | | 0.08 | | 0 |
| 67. | rs139191072 | | Intronic | | G>T | CYP2C18 | | - | | | 0 | 0.148 | | 0.189 | 0.03 | 0.1 | | 0.17 | | 0 |
| 68. | rs149917524 | | Intronic | | G>A | CYP2C18 | | - | | | 0 | 0.461 | | 0.273 | 0 | 2.22 | | 0.25 | | 6.9 |
| 69. | rs41286880 | | Exonic - Missense (R335Q) | | G>A | CYP2C18 | | - | | | 0 | 1.922 | | 2.092 | 0.402 | 0.09 | | 0.75 | | 0.95 |
| 70. | rs41286882 | | Exonic - Missense (C338R) | | T>C | CYP2C18 | | - | | | 0 | 0.2425 | | 0.2634 | 0.07 | 0 | | 0.08 | | 0.1 |
| 71. | rs79500998 | | Exonic - Missense (R442C) | | C>T | CYP2C18 | | - | | | 0 | 0.009 | | 0.003 | 0 | 0.18 | | 0 | | 0 |
| 72. | rs41286884 | | Exonic - Missense (R478H) | | G>A | CYP2C18 | | - | | | 0 | 1.21 | | 1.35 | 0.26 | 0 | | 0 | | 0.2 |
| 73. | rs41291552 | | Intronic | | C>T | CYP2C18 | | - | | | 0 | 1.81 | | 1.98 | 0.19 | 0 | | 0.33 | | 0.58 |
| 74. | rs55752064 | | Exonic - Missense (L17P) | | T>C | CYP2C19 | | CYP2C19*14 | | | 0 | 0 | | 0 | 0 | 0 | | 0 | | 0 |
| 75. | rs1564656981 | | Exonic - Missense (K28I) | | A>T | CYP2C19 | | - | | | 0 | N/K | | N/K | N/K | N/K | | N/K | | N/K |
| 76. | rs1564657013 | | Exonic - Missense (S51G) | | A>G | CYP2C19 | | - | | | 0 | N/K | | N/K | N/K | N/K | | N/K | | N/K |
| 77. | rs572853437 | | Exonic - Missense (T55S) | | C>A | CYP2C19 | | - | | | 0 | 0.06 | | 0 | 0 | 0 | | 0.33 | | 0 |
| 78. | rs148954322 | | Intronic | | G>T | CYP2C19 | | - | | | 0 | 0.142 | | 0.186 | 0 | 0 | | 1.83 | | 0.2 |
| 79. | rs199904246 | | Intronic | | C>T | CYP2C19 | | - | | | 0 | 0.447 | | 0.546 | 0.14 | 0 | | 0.17 | | 0.5 |
| 80. | rs545579039 | | Intronic | | A>G | CYP2C19 | | - | | | 0 | 0.233 | | 0.298 | 0.04 | 0 | | 0 | | 0.2 |
| 81. | rs185348086 | | Intronic | | C>T | CYP2C19 | | - | | | 0 | 0.17 | | 0 | 0 | 0.85 | | 0.08 | | 0 |
| 82. | rs187921622 | | Intronic | | C>A | CYP2C19 | | - | | | 0 | 0.14 | | 0 | 0 | 0.77 | | 0 | | 0 |
| 83. | rs145328984 | | Exonic - Missense (R73C) | | C>A | CYP2C19 | | - | | | 0 | 0.02 | | 0 | 0.08 | 0 | | 0 | | 0 |
| 84. | rs28399505 | | Exonic - Missense (M74T) | | T>C | CYP2C19 | | - | | | 0 | 0.08 | | 0 | 0.4 | 0.43 | | 0 | | 0 |
| 85. | rs1564660997 | | Exonic - Missense (H78Y) | | C>T | CYP2C19 | | - | | | 0 | N/K | | N/K | N/K | N/K | | N/K | | N/K |
| 86. | rs118203756 | | Exonic - Missense (G91R) | | G>C | CYP2C19 | | - | | | 0 | 0.0021 | | 0 | 0 | 0.1 | | 0 | | 0 |
| 87. | rs1288601658 | | Exonic - Missense (H99L) | | A>T | CYP2C19 | | - | | | 0 | 0.0007 | | 0 | 0 | 0 | | 0 | | 0.007 |
| 88. | rs545642100 | | Exonic - Missense (A106G) | | C>G | CYP2C19 | | - | | | 0 | 0.03 | | 0 | 0.11 | 0 | | 0 | | 0 |
| 89. | rs41291556 | | Exonic - Missense (W120R) | | T>C | CYP2C19 | | CYP2C19*8 | | | 0 | 0.11 | | 0.3 | 0.06 | 0 | | 0.08 | | 0 |
| 90. | rs72552267 | | Exonic - Missense (R132Q) | | G>A | CYP2C19 | | - | | | 0 | 0.033 | | 0.03 | 0.012 | 0.06 | | 0 | | 0.069 |
| 91. | rs17884712 | | Exonic - Missense (R144H) | | G>A | CYP2C19 | | CYP2C19*9 | | | 0 | 0.31 | | 0.002 | 1.01 | 0 | | 0 | | 0.2 |
| 92. | rs58973490 | | Exonic - Missense (R150H) | | G>A | CYP2C19 | | - | | | 0 | 0.364 | | 0.408 | 0.14 | 0 | | 0 | | 0 |
| 93. | rs140278421 | | Exonic - Missense (R186H) | | G>A | CYP2C19 | | - | | | 0 | 0.03 | | 0 | 0.06 | 0 | | 0 | | 0.1 |
| 94. | rs370803989 | | Exonic - Missense (D188N) | | G>A | CYP2C19 | | - | | | 0 | 0.005 | | 0.007 | 0.002 | 0 | | 0 | | 0.007 |
| 95. | rs4986893 | | Exonic - Nonense (W212Ter) | | G>A | CYP2C19 | | CYP2C19*3 | | | 0 | 1.31 | | 0 | 0.22 | 5.38 | | 1.41 | | 0 |
| 96. | rs200936950 | | Intronic | | G>A | CYP2C19 | | - | | | 0 | 0.03 | | 0 | 0 | 0 | | 0 | | 0.2 |
| 97. | rs59162501 | | Intronic | | A>G | CYP2C19 | | - | | | 0 | 0.02 | | 0.08 | 0 | 0 | | 0 | | 0 |
| 98. | rs6413438 | | Exonic - Nonsense (P227L) | | C>T | CYP2C19 | | CYP2C19*10 | | | 0 | 0.05 | | 0 | 0.11 | 0 | | 0 | | 0.1 |
| 99. | rs72558185 | | Exonic - Missense (E241G) | | D/I | CYP2C19 | | - | | | 0 | N/K | | N/K | N/K | N/K | | N/K | | N/K |
| 100. | rs375781227 | | Exonic - Missense (D256N) | | G>A | CYP2C19 | | - | | | 0 | 0.0007 | | 0 | 0 | 0.03 | | 0 | | 0 |
| 101. | rs577255883 | | Exonic - Missense (D262N) | | G>A | CYP2C19 | | - | | | 0 | 0.05 | | 0 | 0 | 0 | | 0.25 | | 0 |
| 102. | rs778258371 | | Exonic - Missense (M271I) | | G>A | CYP2C19 | | - | | | 0 | 0.0016 | | 0 | 0 | 0.006 | | 0 | | 0.005 |
| 103. | rs72558186 | | splice donor variant | | T>C | CYP2C19 | | CYP2C19*7 | | | 0 | 0.0005 | | 0.001 | 0 | 0 | | 0 | | 0 |
| 104. | rs143138959 | | Intronic | | C>A | CYP2C19 | | - | | | 0 | 0.97 | | 0 | 0 | 5.13 | | 0.17 | | 0 |
| 105. | rs373408094 | | Intronic | | C>T | CYP2C19 | | - | | | 0 | 0.16 | | 0.16 | 0 | 0.68 | | 0 | | 0 |
| 106. | rs139840199 | | Intronic | | A>G | CYP2C19 | | - | | | 0 | 0.45 | | 0 | 0.11 | 2.22 | | 0.08 | | 0 |
| 107. | rs528218818 | | Intronic | | T>G | CYP2C19 | | - | | | 0 | 1.34 | | 0.89 | 1.36 | 2.38 | | 1.2 | | 0.6 |
| 108. | rs192289355 | | Intronic | | T>C | CYP2C19 | | - | | | 0 | 0.44 | | 0 | 0 | 2.39 | | 0 | | 0 |
| 109. | rs551320091 | | Intronic | | C>T | CYP2C19 | | - | | | 0 | 0.33 | | 0 | 0 | 0 | | 1.75 | | 0 |
| 110. | rs140554854 | | Intronic | | G>T | CYP2C19 | | - | | | 0 | 0.14 | | 0 | 0 | 0.77 | | 0 | | 0 |
| 111. | rs555752933 | | Intronic | | G>A | CYP2C19 | | - | | | 0 | 0.76 | | 0 | 1.97 | 1.09 | | 0 | | 0.1 |
| 112. | rs575616160 | | Intronic | | C>A | CYP2C19 | | - | | | 0 | 0.52 | | 0 | 1.97 | 0 | | 0 | | 0 |
| 113. | rs544345502 | | Intronic | | A>G | CYP2C19 | | - | | | 0 | 0.52 | | 0 | 1.97 | 0 | | 0 | | 0 |
| 114. | rs535259152 | | Intronic | | C>A | CYP2C19 | | - | | | 0 | 0.88 | | 1.69 | 0.15 | 0.99 | | 0 | | 2.2 |
| 115. | rs548874926 | | Intronic | | T>A | CYP2C19 | | - | | | 0 | 0.88 | | 1.69 | 0.15 | 0.99 | | 0 | | 2.2 |
| 116. | rs190777341 | | Intronic | | C>G | CYP2C19 | | - | | | 0 | 0.97 | | 0 | 0 | 4.56 | | 0.17 | | 0 |
| 117. | rs575923433 | | Intronic | | C>T | CYP2C19 | | - | | | 0 | 0.33 | | 0 | 0 | 0 | | 1.75 | | 0 |
| 118. | rs559628884 | | Exonic - Missense (N277K) | | C>A | CYP2C19 | | - | | | 0 | 0.03 | | 0 | 0 | 0.17 | | 0 | | 0 |
| 119. | rs539248491 | | Intronic | | A>G | CYP2C19 | | - | | | 0 | 0.02 | | 0 | 0 | 0 | | 0.08 | | 0 |
| 120. | rs200003088 | | Intronic | | G>A | CYP2C19 | | - | | | 0 | 0.02 | | 0.08 | 0 | 0 | | 0 | | 0 |
| 121. | rs566054687 | | Intronic | | G>A | CYP2C19 | | - | | | 0 | 0.1 | | 0.3 | 0 | 0.2 | | 0 | | 0 |
| 122. | rs555356783 | | Intronic | | C>A | CYP2C19 | | - | | | 0 | 0.41 | | 1.34 | 0.06 | 0.17 | | 0 | | 0.6 |
| 123. | rs186575758 | | Intronic | | G>T | CYP2C19 | | - | | | 0 | 0.02 | | 0 | 0.06 | 0 | | 0 | | 0 |
| 124. | rs75105257 | | Intronic | | T>C | CYP2C19 | | - | | | 0 | 1.78 | | 0 | 0.11 | 1.45 | | 0 | | 9.7 |
| 125. | rs138142612 | | Exonic - Missense (R329H) | | G>A | CYP2C19 | | - | | | 0 | 0.005 | | 0.009 | 0 | 0 | | 0 | | 0 |
| 126. | rs118203757 | | Exonic - Missense (R335Q) | | G>A | CYP2C19 | | - | | | 0 | 0.02 | | 0 | 0.06 | 0 | | 0 | | 0 |
| 127. | rs201132803 | | Exonic - Missense (M345K) | | T>A | CYP2C19 | | - | | | 0 | 0.02 | | 0.08 | 0 | 0 | | 0 | | 0 |
| 128. | rs144036596 | | Exonic - Missense (D360N) | | G>A | CYP2C19 | | - | | | 0 | 0.03 | | 0 | 0 | 0 | | 0 | | 0.2 |
| 129. | rs550527959 | | Exonic - Missense (D360V) | | A>T | CYP2C19 | | - | | | 0 | 0.02 | | 0 | 0 | 0.09 | | 0 | | 0 |
| 130. | rs771120274 | | Exonic - Nonsense (Y379Ter) | | C>A | CYP2C19 | | - | | | 0 | 0.017 | | 0 | 0 | 0 | | 0 | | 0.124 |
| 131. | rs185136199 | | Intronic | | C>G | CYP2C19 | | - | | | 0 | 0.02 | | 0.08 | 0 | 0 | | 0 | | 0 |
| 132. | rs55948420 | | Exonic - Missense (V394M) | | G>A | CYP2C19 | | - | | | 0 | 0.0007 | | 0 | 0 | 0 | | 0 | | 0.03 |
| 133. | rs144056033 | | Exonic - Missense (M406V) | | A>G | CYP2C19 | | - | | | 0 | 0.02 | | 0 | 0.06 | 0 | | 0 | | 0 |
| 134. | rs527499296 | | Exonic - Missense (E415D) | | A>C | CYP2C19 | | - | | | 0 | 0.02 | | 0 | 0.06 | 0 | | 0 | | 0 |
| 135. | rs56337013 | | Exonic - Missense (R433W) | | C>T | CYP2C19 | | CYP2C19*5 | | | 0 | 0.0007 | | 0.001 | 0 | 0 | | 0 | | 0 |
| 136. | rs5787121 | | N/K | | D/I | CYP2C19 | | - | | | 0 | N/K | | N/K | N/K | N/K | | N/K | | N/K |
| 137. | rs192154563 | | Exonic - Missense (R442C) | | C>G | CYP2C19 | | - | | | 0 | 0.02 | | 0 | 0.06 | 0 | | 0 | | 0 |
| 138. | rs118203759 | | Exonic - Missense (F448L) | | C>A | CYP2C19 | | - | | | 0 | 0 | | 0 | 0 | 0 | | 0 | | 0 |
| 139. | rs375283723 | | Exonic - Synonymous (D466D) | | C>T | CYP2C19 | | - | | | 0 | 0.02 | | 0.004 | 0 | 0 | | 0 | | 0 |
| 140. | rs28399514 | | Exonic - Synonymous (P480P) | | G>A | CYP2C19 | | - | | | 0 | 0.036 | | 0.05 | 0 | 0 | | 0 | | 0.035 |
| 141. | rs55640102 | | Exonic – Stop lost (Ter491C) | | A>C | CYP2C19 | | CYP2C19*12 | | | 0 | 0.0432 | | 0.0435 | 0.135 | 0 | | 0 | | 0 |

*MAF of *CYP2C19* and *CYP2C18* variants related to global, European, African, East Asian, South Asian, and Latin American ethnic groups were obtained from <https://www.ncbi.nlm.nih.gov/snp/>. N/K: Not known

| Variant | Star allele | MAF, % | | | | | | | | | | | | |  |
| --- | --- | --- | --- | --- | --- | --- | --- | --- | --- | --- | --- | --- | --- | --- | --- |
|  |  | **current study** | **Saudi Arabia* [6]** | **Saudi Arabia****  **[24]** | **Bahrain**  **[25]** | **Kuwait**  **[25]** | **Tunis**  **[25]** | **Iraq**  **[26]** | **Egypt**  **[27]** | **Israel**  **[28]** | **Palestine**  **[29]** | **Lebanon**  **[30]** | **Jordan**  **[31]** | **Iran**  **[32]** | |
| rs4244285 | CYP2C19*2 | NM | 8.2 | 9.9 | 14.6 | 10.5 | 9 | 15.15 | 12.6 | 15 | 15.45 | 26.6 | 9.8 | 13.3 | |
| rs4986893 | CYP2C19*3 | 0 | 0 | 0.1 | NM | NM | 0 | 0.22 | 0.25 | 1 | 2.27 | NM | NM | 0 | |
| rs17878459 | CYP2C19*3 | 0.67 | NM | 0 | NM | NM | NM | NM | NM | NM | NM | NM | NM | NM | |
| rs28399504 | CYP2C19*4 | NM | 0 | 0 | NM | NM | NM | NM | NM | NM | NM | NM | NM | NM | |
| rs56337013 | CYP2C19*5 | 0 | NM | 0 | NM | NM | NM | NM | NM | NM | NM | NM | NM | NM | |
| rs2558184 | CYP2C19*6 | NM | 0 | 0.2 | NM | NM | NM | NM | 0 | NM | NM | NM | NM | NM | |
| rs72558186 | CYP2C19*7 | 0 | 0 | 0 | NM | NM | NM | NM | NM | NM | NM | NM | NM | NM | |
| rs41291556 | CYP2C19*8 | 0 | NM | 0.1 | NM | NM | NM | NM | 0 | NM | NM | NM | NM | NM | |
| rs17884712 | CYP2C19*9 | 0 | NM | 0.2 | NM | NM | NM | NM | NM | NM | NM | NM | NM | NM | |
| rs6413438 | CYP2C19*10 | 0 | NM | 0 | NM | NM | NM | NM | 0 | NM | NM | NM | NM | NM | |
| rs55640102 | CYP2C19*12 | 0 | NM | 0 | NM | NM | NM | NM | NM | NM | NM | NM | NM | NM | |
| rs17879685 | CYP2C19*13 | 0.13 | NM | 0.1 | NM | NM | NM | NM | NM | NM | NM | NM | NM | NM | |
| rs55752064 | CYP2C19*14 | 0 | NM | 0 | NM | NM | NM | NM | NM | NM | NM | NM | NM | NM | |
| rs17882687 | CYP2C19*15 | 0 | NM | 0.1 | NM | NM | NM | NM | NM | NM | NM | NM | NM | NM | |
| rs12248560 | CYP2C19*17 | 20.99 | 26.9 | 25.7 | 27.3 | 25 | 21.3 | 19.45 | 17 | NM | NM | NM | 28.7 | 21.6 | |
| rs3758581 | CYP2C19*1B | NM | NM | 5.5 | NM | NM | NM | NM | NM | NM | NM | NM | NM | NM | |

**Table S4 Comparison of *CYP2C19* star allele frequencies among the Middle Eastern populations.**

NM: not measured. *A Saudi study published in 2013. **A Saudi study published in 2015.
